# Text Knows What, Tables Know When: Clinical Timeline Reconstruction via Retrieval-Augmented Multimodal Alignment

**Published:** 2026-05-14

**Authors:** Sayantan Kumar, Shahriar Noroozizadeh, Juyong Kim, Jeremy C. Weiss

**Affiliations:** Division of Intramural Research, National Library of Medicine, National Institutes of Health, Bethesda, MD, USA; Machine Learning Department, School of Computer Science, Heinz College of Information Systems and Public Policy Management, Carnegie Mellon University, Pittsburgh, PA, USA; Machine Learning Department, School of Computer Science, Carnegie Mellon University, Pittsburgh, PA, USA; Division of Intramural Research, National Library of Medicine, National Institutes of Health, Bethesda, MD, USA

## Abstract

Reconstructing precise clinical timelines is essential for modeling patient trajectories and forecasting risk in complex, heterogeneous conditions like sepsis. While unstructured clinical narratives offer semantically rich and contextually complete descriptions of a patient’s course, they often lack temporal precision and contain ambiguous event timing. Conversely, structured electronic health record (EHR) data provides precise temporal anchors but misses a substantial portion of clinically meaningful events. We introduce a retrieval-augmented multimodal alignment framework that bridges this gap to improve the temporal precision of absolute clinical timelines extracted from text. Our approach formulates timeline reconstruction as a graph-based multistep process: it first extracts central anchor events from narratives to build an initial temporal scaffold, places non-central events relative to this backbone, and then calibrates the timeline using retrieved structured EHR rows as external temporal evidence. Evaluated using instruction-tuned large language models on the i2m4 benchmark spanning MIMIC-III and MIMIC-IV, our multimodal pipeline consistently improves absolute timestamp accuracy (AULTC) and improves temporal concordance across nearly all evaluated models over unimodal text-only reconstruction, without compromising event match rates. Furthermore, our empirical gap analysis reveals that 34.8% of text-derived events are entirely absent from tabular records, demonstrating that aligning these modalities can produce a more temporally faithful and clinically informative reconstruction of patient trajectories than either source alone.

## 1. Introduction

Sepsis, as defined by the Third International Consensus Definitions for Sepsis (Sepsis-3), remains a central clinical target for both trial eligibility and computational phenotyping (Kyriazopoulou et al., 2021; Seymour et al., 2019). At the same time, sepsis is not a single uniform disease process; in critical care it is better understood as a heterogeneous clinical trajectory shaped by diverse infectious sources, anatomical sites, and comorbid conditions. This makes time especially important: understanding when symptoms emerge, how physiologic deterioration unfolds, and how the patient trajectory evolves is essential for meaningful risk forecasting in sepsis. The importance of temporal structure is increasingly reflected across the literature, including early-warning systems (Henry et al., 2022), studies showing that predictive utility varies with timing (Kamran et al., 2024), and work on temporal characterization of sepsis subtypes and progression (Noroozizadeh et al., 2023).

This growing emphasis on time in sepsis also highlights an important methodological gap. Critical care repositories such as MIMIC-III (Johnson et al., 2016) and MIMIC-IV (Johnson et al., 2023) come closest to the multimodal EHR setting encountered in real hospital care, pairing structured time-series data with narrative clinical documentation. These two sources offer complementary strengths for timeline construction. Structured data provides relatively precise temporal evidence through laboratory values, medications, procedures, and physiologic measurements. In contrast, clinical narratives often contain semantically richer and more contextually complete descriptions of the patient course and often contain early indicators of deterioration not yet reflected in quantitative metrics. For example, a clinician’s note describing “increasing lethargy and peripheral mottling” may appear before a rise in Sequential Organ Failure Assessment (SOFA) scores, providing an early signal of impending cardiovascular collapse. In addition, discrepancies between narrative observations and measured parameters (e.g., documented oliguria despite stable creatinine) may reveal subtypes of organ dysfunction with distinct prognostic implications. More generally, prior work has shown that structured and unstructured clinical data are complementary for phenotyping and prediction (Moldwin et al., 2021; Seinen et al., 2025).

Recent studies point to both the promise and the difficulty of this problem. Multimodal annotation of discharge summaries aligned with structured EHR data demonstrated that access to both modalities can improve the precision of inpatient event timelines, highlighting the value of structured rows as temporal evidence when aligned with the narrative record (Frattallone-Llado et al., 2024). At the same time, text-only timeline reconstruction remains challenging in realistic EHR-style notes, where dense event structure, abbreviations, and semi-structured formatting make both event recovery and time assignment difficult (Noroozizadeh and Weiss, 2026; Noroozizadeh et al., 2026). Together, these findings motivate more explicit alignment between text-based and tabular approaches, with the goal of improving temporal precision while preserving the richer event content available in narrative text.

In this work, we build on this motivation by studying how retrieved structured EHR evidence can be aligned with text-derived event timelines to improve the temporal precision of reconstructed patient trajectories. Our approach uses retrieval-augmented multimodal integration to bring structured rows into the timeline reconstruction process rather than treating text and tabular data as separate views. To make this alignment effective, we implement it through a graph-based multistep pipeline. The pipeline first identifies temporally informative central events, uses them to build an initial scaffold for the patient trajectory, and then places additional events relative to that scaffold before refining the timeline with retrieved structured evidence. This decomposition reflects a clinically natural distinction between events that define the backbone of an encounter and events whose timing is better interpreted relative to that backbone. Using this framework, we study whether and where multimodal alignment improves over unimodal text-only timeline reconstruction. In particular, we examine the effects of introducing structured evidence at different stages of the graph, evaluate whether gains are concentrated in temporal metrics rather than event recovery itself, and analyze what kinds of prognostically relevant information are present in text but missing or delayed in tabular data.

**Figure 1:**
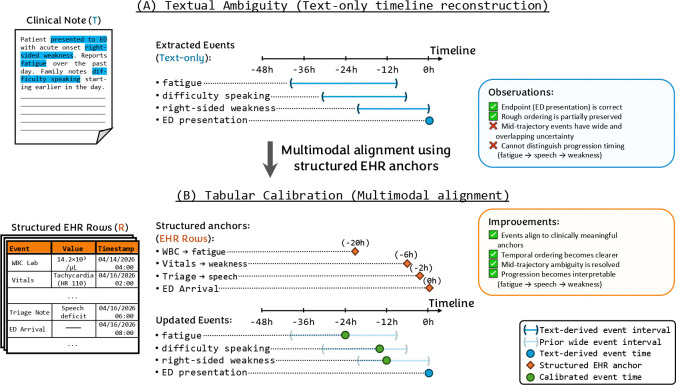
Why multimodal alignment can improve temporal precision in clinical timeline reconstruction. (**A**) In text-only reconstruction, events can often be recovered and placed in roughly plausible order, but intermediate events may retain wide or overlapping uncertainty intervals, making progression difficult to interpret. (**B**) Retrieved structured EHR rows provide temporally precise anchors that calibrate these text-derived events, improving temporal ordering and narrowing ambiguity while preserving the richer event content from the narrative.

Figure 1 illustrates the core intuition behind our approach. Text-only reconstruction can recover clinically meaningful events from narrative notes, but the timing of intermediate events often remains ambiguous. Retrieved structured EHR rows provide sharper temporal anchors for subsets of these events, allowing the reconstructed trajectory to be temporally calibrated without discarding the richer semantic content available in narrative text.

### Contributions.

In summary, our work makes the following contributions: (i) **RAG-based multimodal integration for improved temporal precision:** We develop a retrieval-augmented multimodal framework that aligns text-derived event timelines with structured EHR rows from MIMIC-III and MIMIC-IV, and show that this integration improves temporal precision over unimodal text-only reconstruction; (ii) **Multistep graph-based timeline reconstruction:** We develop a graph-based multistep pipeline for reconstructing absolute clinical timelines from unstructured text and structured EHR data. The pipeline uses central events as temporal anchors, then places non-central events relative to that scaffold, enabling more precise timeline reconstruction than a single-pass formulation; (iii) **Stage-specific integration of structured evidence:** We compare variants that calibrate the central timeline only, the final timeline only, both stages, or neither stage, thereby isolating how retrieved structured evidence contributes to timeline quality; (iv) **Information missing from tabular data:** We provide an empirical analysis of clinically relevant events that are absent, delayed, or semantically compressed in structured tabular data, motivating why unstructured text is necessary for constructing clinically meaningful patient trajectories; and (v) **When multimodal alignment helps most:** We characterize performance across event types and timestamp-certainty settings, showing that structured data is especially useful as a temporal calibrator when event timing cannot be directly inferred from text.

### Generalizable Insights about Machine Learning in the Context of Healthcare

This work suggests that, in healthcare, access to the best temporal information available is often as important as access to the best predictive features. Clinical timeline construction should therefore be viewed not as a text-only extraction task or a tabular-only retrieval task, but as an alignment problem between complementary sources of temporal evidence. In settings such as MIMIC-III and MIMIC-IV, narrative text often provides the most complete account of the patient course, including early symptoms, contextual assessments, and progression, whereas structured records often provide sharper temporal anchors for subsets of events. This distinction matters because retrospective clinical narratives are frequently organized for communication rather than chronology, which can obscure temporal dependencies and introduce leakage when they are used directly for downstream modeling. By reconstructing explicit patient timelines, multimodal timeline methods can transform retrospective records into longitudinally structured representations that are better suited to risk forecasting, causal analyses, and temporal decision support. Although this study is motivated by sepsis, the underlying principle is broader: whenever structured and unstructured clinical data are both available, aligning them can yield a temporally more faithful patient trajectory than either source alone. Even in text-only settings, decomposing the task around temporally informative anchor events may still improve timeline quality.

## 2. Related Work

Prior work in clinical temporal information extraction has focused primarily on recovering temporal relations between concepts in text rather than assigning events explicit timestamps. The i2b2 temporal relation framework is the canonical example of this line of work, where the task is to determine whether one concept occurs before, after, or overlaps another (Sun et al., 2013). While this formulation has been highly influential, it does not directly yield the timestamped patient trajectories needed for downstream temporal modeling. Subsequent work moved closer to absolute event timing (Leeuwenberg and Moens, 2020; Frattallone-Llado et al., 2024), but these studies were conducted in relatively constrained settings, such as excerpted reports, pre-specified spans, or small annotated cohorts. Our work builds on this literature by focusing on absolute clinical timeline reconstruction in realistic EHR-style records and by studying how structured evidence can be used to improve temporal precision.

Among prior work that has addressed timeline construction more directly, Frattallone-Llado et al. (2024) is particularly relevant because it showed that aligning discharge summaries with structured EHR data can improve the precision of inpatient event timelines. Building on the insight of Frattallone-Llado et al. (2024) that structured EHR data can improve timeline precision when aligned with discharge summaries, we study how such structured evidence can be integrated directly into a retrieval-augmented multistep reconstruction pipeline for absolute clinical timeline generation. More recent work extended timeline reconstruction into large-scale text-only settings through textual time-series construction and forecasting-oriented pipelines (Noroozizadeh and Weiss, 2026; Noroozizadeh et al., 2025, 2026). These works demonstrate that narrative text can support clinically meaningful temporal reconstruction, but they are fundamentally unimodal. In contrast, our work focuses on MIMIC-III and MIMIC-IV (Johnson et al., 2016, 2023), where structured event streams and clinical narratives coexist within the same record. This makes our setting more suitable for studying multimodal alignment between text-derived events and structured temporal evidence. Methodologically, our contribution also differs from single-step reconstruction approaches by introducing a graph-based multistep pipeline in which central events serve as temporal anchors for subsequent timeline refinement.

Large language models have increasingly been used for clinical text understanding, with promising results in tasks such as summarization and other forms of medical language processing (Van Veen et al., 2024). At the same time, recent evidence suggests that medical adaptation does not always outperform strong foundation models (Jeong et al., 2024). This is relevant to our setting because timeline reconstruction requires both semantic interpretation of narrative text and flexible temporal reasoning over heterogeneous evidence sources. Our work is not primarily an LLM benchmarking study. Instead, it uses capable instruction-tuned foundation models within a structured retrieval-augmented pipeline, where LLM-based reasoning is combined with retrieved structured EHR rows to reconstruct temporally precise patient trajectories.

## 3. Methods

### 3.1. Task formulation

Given a clinical text document T, we extract a textual time-series $S=\left\{\left(e_{1},t_{1}\right),\left(e_{2},t_{2}\right),\ldots ,\left(e_{n},t_{n}\right)\right\}$, where each $e_{i}$ denotes a clinical event and each $t_{i}\in R$ denotes its timestamp in hours relative to a case-specific reference time. When admission is explicitly described, the reference time is hospital admission (t=0); otherwise, we use the earliest documented clinical encounter or presentation in the narrative. Events before the reference time receive negative timestamps, and events after it receive positive timestamps.

A clinical event is any health-related mention that is semantically interpretable on its own and directly relevant to the patient’s course. This includes symptoms and signs, diagnoses, procedures and diagnostic tests, treatments and medication administrations, major clinical states, outcomes, pertinent negatives, and termination events. Demographic attributes mentioned in the narrative, such as age and sex, are also represented as events with timestamp t=0. We exclude contextual text that does not describe the index patient.

Our formulation follows Wang and Weiss (2025); Noroozizadeh and Weiss (2026) and differs from i2b2-style clinical concept annotation of Uzuner et al. (2011); Sun et al. (2013) in three ways. First, event spans may extend beyond short phrases when additional context is needed for clinical specificity. Second, conjunctive mentions are split into separate events when doing so improves clarity. Third, semantic modifiers that alter the clinical status of a mention—including negation, uncertainty, and intent—are preserved because they materially affect both event interpretation and temporal placement.

Temporal assignment is defined at the event level and aims to capture event start time whenever such a start can be inferred. Natural-language time expressions are normalized into hours relative to the reference time: coarse expressions such as “hospital day 2” are converted using 24-hour increments, and interval expressions are represented by their start when a start is recoverable. For event mentions that do not provide a recoverable onset, we assign the time at which the condition is first documented or contextually asserted in the narrative. More generally, vague temporal phrases are resolved to approximate offsets using narrative ordering and nearby temporal cues. This yields a temporally explicit representation of the patient course that is better suited to downstream modeling than relation-only temporal annotations.

### 3.2. Data and Gold Standard Processing

Our evaluation uses the publicly available absolute timeline annotations introduced by Frattallone-Llado et al. (2024). These annotations were derived from physician review of 20 discharge summaries originally annotated under i2b2-style concept and temporal guidelines, with the additional assignment of interval-valued timestamps. Fifteen summaries come from the i2b2 subset of MIMIC-II/III and five from MIMIC-IV; we refer to this combined benchmark as i2m4. For analysis, we use the probabilistic annotations only and take the lower-bound mean time of each annotated interval as the event timestamp, since it most naturally represents the earliest time at which the finding may first be observed.

#### Reformatting of gold standard annotations.

Although i2m4 provides expert timeline annotations, the released annotation scheme is concept-centric and does not fully align with the standalone event representation used in our textual time-series (TTS) formulation. To make comparison with LLM-generated TTS outputs as fair as possible, we therefore convert the gold standard into a TTS-compatible format using a reasoning model that operates only on the released absolute timeline annotations, not on the raw discharge summaries. This preserves the underlying physician-provided temporal information while adapting the reference to the representational target used by our reconstruction pipeline. Concretely, we consider three versions of the manual reference: v1, the original released annotations; v2, a minimally cleaned rule-based version; and v3, the final rule-based plus LLM-reformatted version, which we manually verify to ensure no hallucinated content is introduced and that all outputs remain grounded in the original annotations. To make this formatting choice transparent, we report results for v3 in the main body and provide side-by-side comparisons across all three versions in Appendix D and G, where we also detail the reformatting procedure and analysis.

### 3.3. Multistep workflow for timeline reconstruction

**Figure 2:**
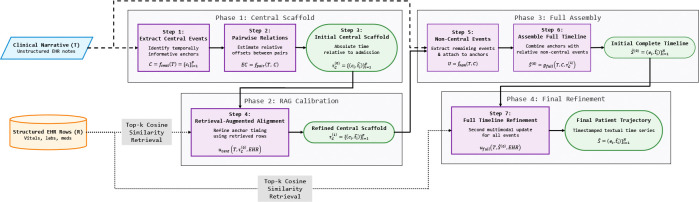
Overview of the proposed multistep retrieval-augmented multimodal timeline reconstruction pipeline. Starting from a clinical narrative, T, the method first extracts temporally informative central events and estimates pairwise temporal relations to build an initial central scaffold. Retrieved structured EHR rows, R, are then used to calibrate this scaffold. The method next extracts non-central events relative to the central anchors, ssembles an initial complete timeline, and performs a second retrieval-augmented refinement over the full event set. This yields a final timestamped textual time-series that combines the semantic richness of narrative text with the temporal precision of structured EHR evidence.

An overview of the full pipeline is shown in Figure 2. Let T denote a clinical narrative and let $R={\left\{r_{m}\right\}}_{m=1}^{M}$ denote the structured EHR rows associated with the same encounter, where each row $r_{m}=\left(\nu_{m},x_{m},s_{m}\right)$ contains an event name $\nu_{m}$, an observed value $x_{m}$, and a timestamp $s_{m}\in R$ in hours relative to admission. Our goal is to reconstruct a textual time-series $\overset{ˆ}{S}={\left\{\left(e_{i},{\overset{ˆ}{t}}_{i}\right)\right\}}_{i=1}^{N}$, where each $e_{i}$ is a clinical event extracted from the narrative and ${\overset{ˆ}{t}}_{i}$ is its predicted timestamp.

Rather than predicting $\overset{ˆ}{S}$ in a single pass, we decompose the problem into four phases: central scaffold construction, scaffold calibration using retrieved structured evidence, full timeline assembly, and final timeline refinement. This reflects our central hypothesis that structured EHR data is most useful as temporal support for a text-derived event trajectory rather than as a replacement for narrative event content.

#### Step 1: Central-event extraction.

From the narrative T, we first extract a set of temporally informative central events,

$$C=f_{\text{cent}}\left(T\right)=\left\{c_{1},\ldots ,c_{P}\right\}.$$

These events serve as temporal anchors for subsequent reconstruction steps (Appendix A.1).

#### Step 2: Pairwise temporal relations among central events.

For each relevant ordered pair of central events, we estimate a relative temporal offset,

$$E_{C}=f_{pair}(T,C)=\left\{\left(c_{i},c_{j},\Delta_{ij},q_{ij}\right)\right\},$$

where the ordered pair $\left(c_{i},c_{j}\right)$ denotes a directed temporal relation from $c_{i}$ to $c_{j}$, and

$$\Delta_{ij}=t_{j}-t_{i}$$

is the predicted offset in hours from $c_{i}$ to $c_{j}$. Thus, a positive $\Delta_{ij}$ indicates that $c_{j}$ occurs after $c_{i}$, while a negative $\Delta_{ij}$ indicates that $c_{j}$ occurs before $c_{i}$. The term $q_{ij}$ denotes an associated confidence score. This induces a central-event graph

$$G_{C}=\left(V_{C},E_{C}\right),\;V_{C}=C,$$

whose nodes are central events and whose edges encode pairwise temporal constraints (Appendix A.2).

#### Step 3: Initial central timeline reconstruction.

Given the central-event graph and the source narrative, we reconstruct an initial central timeline,

$$\tau_{C}^{\left(0\right)}=g_{\text{cent}}\left(T,G_{C}\right)={\left\{\left(c_{i},{\overset{ˆ}{t}}_{i}^{\left(0\right)}\right)\right\}}_{i=1}^{P}.$$

This step converts relative temporal constraints into an absolute scaffold in hours relative to admission (Appendix A.3).

#### Step 4: Retrieval-augmented calibration of the central scaffold.

To refine the central scaffold, we retrieve the top-k structured EHR rows most relevant to each central event. Let ϕ(·) denote the embedding function used for retrieval. For any event e, define its structured neighborhood as

$${𝒩}_{k}(e;R)={TopK}_{r\in R}\;sim(\phi (e),\phi (r)),$$

where sim(⋅,⋅) is cosine similarity. Using these retrieved neighborhoods, we update the central timeline:

$$\tau_{C}^{\left(1\right)}=u_{\text{cent}}\left(T,\tau_{C}^{\left(0\right)},{\left\{{𝒩}_{k}\left(c_{i};R\right)\right\}}_{i=1}^{P}\right).$$

#### Step 5: Non-central event extraction relative to central events.

We next extract the remaining events together with their attachment to the central-event scaffold:

$$U=f_{non}(T,C)={\left\{\left(u_{\ell },c_{\alpha (\ell )},\delta_{\ell },q_{\ell }\right)\right\}}_{\ell =1}^{L},$$

where $u_{\ell }$ is a non-central event, $c_{\alpha (\ell )}\in C$ is its assigned central event, $\delta_{\ell }$ is its predicted offset in hours relative to that central event, and $q_{\ell }$ is an associated confidence score (Appendix A.4).

#### Step 6: Full timeline reconstruction.

The full event set is then reconstructed by combining the calibrated central scaffold with the relative non-central events:

$${\overset{ˆ}{S}}^{\left(0\right)}=g_{\text{full}}(T,\tau_{C}^{\left(1\right)},U).$$

This yields an initial complete timeline containing both central and non-central events (Appendix A.5).

#### Step 7: Retrieval-augmented refinement of the full timeline.

Finally, we again retrieve structured evidence, now for every event in the reconstructed timeline, and perform a second calibration step:

$$\overset{ˆ}{S}=u_{\text{full}}\left(T,{\overset{ˆ}{S}}^{\left(0\right)},{\left\{{𝒩}_{k}(e;R)\right\}}_{e\in {\overset{ˆ}{S}}^{\left(0\right)}}\right).$$

This second update allows event times to be refined after the full trajectory has been assembled (Appendix A.6).

All operators $f_{\text{cent}}$, $f_{\text{pair}}$, $g_{\text{cent}}$, $u_{\text{cent}}$, $f_{\text{non}}$, $g_{\text{full}}$, and $u_{\text{full}}$ are implemented using an instruction-tuned large language model with structured prompts and constrained output formats (Appendix A). We focus on state-of-the-art instruction-tuned foundation models rather than domain-specific medical LLMs, as recent evidence (Jeong et al., 2024) suggests the former often provide superior zero-shot temporal and semantic reasoning capabilities. The overall workflow is orchestrated using LangChain and LangGraph, with each stage represented as a node in the graph and intermediate timeline states passed between nodes.

#### Baseline and ablation variants.

The default multimodal pipeline applies retrieval-augmented structured calibration at two stages: first to the central-event scaffold through $u_{\text{cent}}$, and then to the reconstructed full timeline through $u_{\text{full}}$. To understand where multimodal evidence is most useful, we evaluate four ablation variants. (i) **Unimodal text-only reconstruction** removes both update steps and serves as the primary baseline. (ii) **Single-step multimodal reconstruction** removes the central/non-central decomposition and directly generates a complete timeline from the narrative with retrieved structured evidence (Appendix A.7). (iii) **Central-only update** applies $u_{\text{cent}}$ but omits $u_{\text{full}}$. (iv) **Final-only update** omits $u_{\text{cent}}$ and applies only $u_{\text{full}}$ after the full event set has been reconstructed.

### 3.4. Evaluation Methodology

We evaluate predicted timelines along two complementary dimensions: *event recovery* and *temporal localization*. Because predicted and reference timelines may differ in length and may not contain identical event strings, we first align predicted events to manually annotated reference events using a recursive best-match procedure adapted from Wang and Weiss (2025); Noroozizadeh and Weiss (2026). This produces a one-to-one alignment between predicted and reference events while accommodating timelines of unequal length. We then quantify event recovery using ***event match rate***, defined as the proportion of reference clinical events that are successfully aligned to a predicted event. Details of the matching algorithm are provided in Appendix B.

Temporal performance is evaluated on the matched subset using two complementary metrics. ***Temporal concordance*** (c-index) measures whether the relative ordering of event times in the predicted timeline agrees with the manually annotated reference. ***Area Under the Log-Time Cumulative Distribution Function (AULTC)*** summarizes absolute timestamp discrepancy on the log-transformed time scale across clinically meaningful time ranges. Together, concordance and AULTC distinguish whether a method preserves event ordering and whether it places events at temporally accurate locations. Accordingly, concordance and AULTC should be interpreted jointly with event match rate, since both temporal metrics are computed only on aligned events. Mathematical definitions are provided in Appendix B.

### 3.5. Additional Sensitivity Analyses

In addition to the main evaluation, we perform a stratified sensitivity analysis over three event-level flags—certain, certain_EHR, and is_central—to understand how pipeline performance varies across different categories of predicted events.

The certain flag is derived from the model-generated confidence scores (e.g., the $q_{ij}$ and $q_{\ell }$ values extracted in Steps 2 and 5) returned with predicted relative timestamps, and serves as an operational proxy for textual temporal certainty. We set certain=1 when the confidence score indicates high certainty (empirically, between 6 and 9 on the model’s output scale), and certain=0 otherwise. The certain_EHR flag indicates that a timestamp is supported or refined using structured EHR evidence. The is_central flag indicates that the event is part of the central-event scaffold in the multistep graph pipeline.

For each flag-defined subset, we report the three main evaluation metrics introduced above—event match rate, temporal concordance, and AULTC—and two additional temporal metrics: ***Anchored Concordance***, which measures relative temporal ordering with respect to a shared set of matched anchor events, and ***Anchored Concordance (Central)***, which restricts this comparison to anchor events drawn from the central-event scaffold. This analysis allows us to distinguish whether some event subsets are easier to recover from text, easier to place in time once recovered, or both. Full per-model results are provided in Appendix E.

### 3.6. Information missing from tabular data

To characterize what clinically relevant information is not well represented in structured EHR data, we perform an auxiliary gap analysis comparing text-derived timelines against tabular records for the same encounters. The analysis uses the best-performing LLM-generated event timelines (from Table 1) as the textual view, MIMIC structured data as the tabular view, and hospital admission time as the shared temporal anchor.

For each textual event, we retrieve a candidate tabular counterpart using embedding-based similarity matching and evaluate it along two dimensions: semantic adequacy and temporal alignment. Semantic adequacy is assessed using retrieval-augmented scoring on a 0–1 scale, while temporal alignment is measured as the absolute difference in hours between the text-derived timestamp and the matched tabular timestamp. Based on these criteria, each event is classified as *well captured*, *complete absence*, *temporal mismatch*, *semantic distance*, or *detail gap*. This analysis quantifies not only whether tabular counterparts exist, but also whether they occur at the right time and preserve the clinical meaning of the original textual event. Full definitions and extended analyses are provided in Appendix C.

## 4. Results

### 4.1. Evaluating quality of clinical timelines

Table 1 summarizes event extraction and temporal localization performance at an event-matching threshold of 0.1 for unimodal and multimodal timeline reconstruction under the v3 gold standard annotations. Side-by-side results for all three annotation versions (v1, v2, and v3) are provided in Appendix G, Table G.1.

Across models, retrieval-augmented multimodal refinement has little effect on event match rate but more often improves temporal quality. Event match rate is unchanged or only marginally different between unimodal and multimodal settings for most models, suggesting that structured EHR evidence does not substantially alter which events are recovered from text. In contrast, AULTC increases for all models under multimodal refinement, while concordance improves for five of seven models, indicating that the main value of structured evidence lies in refining event timing rather than improving event extraction.

The strongest event recovery is achieved by DeepSeek V3.2, which attains an event match rate of 0.588 in both unimodal and multimodal settings. The best temporal performance, however, is obtained by GLM5 in the multimodal setting, which achieves the highest concordance (0.812) and AULTC (0.829) despite a substantially lower event match rate (0.319). These results therefore indicate a trade-off: models that align more events to the manual reference do not necessarily achieve the strongest temporal quality on the matched subset. Because concordance and AULTC are computed only on matched events, these temporal metrics should be interpreted jointly with event match rate rather than in isolation. In particular, stronger temporal performance on the matched subset does not necessarily imply broader event recovery.

**Figure G.1:**
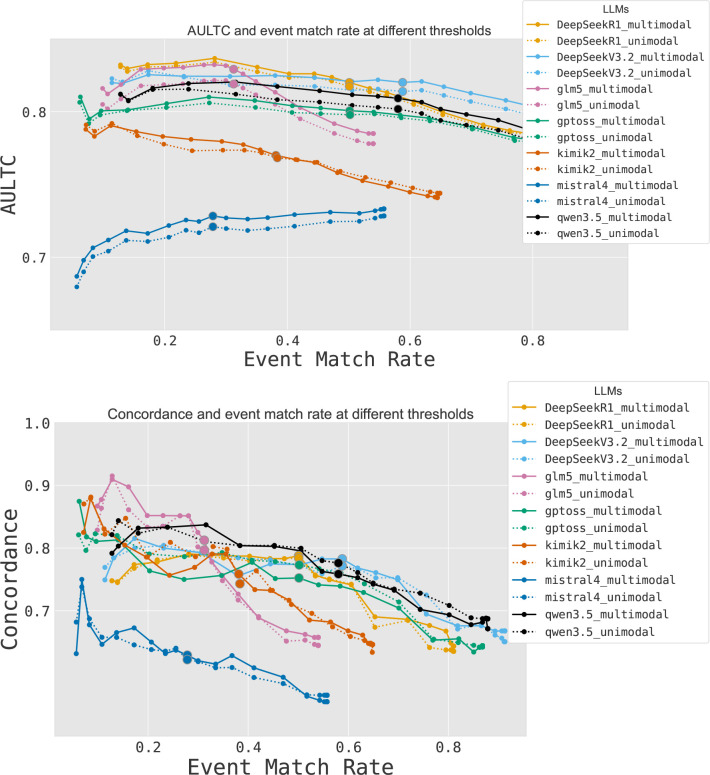
Temporal performance versus event match rate across event-matching thresholds for unimodal and multimodal variants of each model (v3 **version of manual gold standard annotations**). The top panel shows AULTC versus event match rate, and the bottom panel shows temporal concordance versus event match rate.

The threshold-sweep analyses in Appendix G (Figures G.1–G.3) show that these patterns are not specific to a single operating point. Varying the event-matching threshold from 0.01 to 0.50 traces model-specific trade-offs between event recovery and temporal quality across a range of matching stringencies. Across thresholds, multimodal refinement often shifts the frontier upward in AULTC and, for several models, in concordance, with little change in event recovery. Taken together, these analyses reinforce the main conclusion that retrieved structured EHR evidence primarily improves temporal precision rather than event recovery.

### 4.2. Ablation analyses

DeepSeek V3.2 was selected for ablation analysis based on its strongest overall balance between event recovery and temporal performance in the main evaluation (Table 1). Table 2 shows that both the multistep graph decomposition and the stage at which structured evidence is introduced materially affect performance. The single-step formulation performs worst in event recovery, indicating that factorizing timeline reconstruction around central anchor events is beneficial relative to direct one-shot generation. Updating only the final timeline yields the highest event match rate, but not the best temporal quality. In contrast, the default configuration, which calibrates both the central scaffold and the final full timeline, achieves the best concordance and AULTC while maintaining strong event match rate. Central-only updating improves over the single-step baseline and achieves competitive AULTC, but still underperforms the full two-stage update. Taken together, these ablations support the design choice underlying our method: structured evidence is most effective when used both to refine the temporal backbone of the encounter and to recalibrate the expanded full timeline. Additional ablation results for DeepSeek R1 and Qwen3.5, the next best models by overall performance, are provided in Appendix F.

### 4.3. Sensitivity analyses

**Figure 3:**
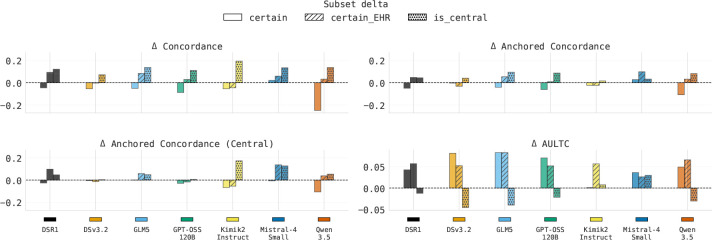
Sensitivity analysis across event subsets. Each bar shows the change in performance between subset = 1 and subset = 0 (i.e., Δ = metric(1) − metric(0)) for each model. We report changes in concordance, anchored concordance, anchored concordance (central), and AULTC for the three flags: certain, certain EHR, and is_central. Positive Δ indicates better performance for the flagged subset. Across all events, the proportion of flagged events is 77.5% for certain=1, 22.5% for certain_EHR=1, and 18.8% for is_central=1.

We next examine how timeline reconstruction performance varies across event subsets defined by temporal certainty, use of structured evidence, and role in the multistep reconstruction pipeline. Specifically, we analyze three event-level flags (as defined in Section 3.5): certain, certain_EHR, and is_central. In addition to event match rate, concordance, and AULTC, we report two anchored temporal metrics—anchored concordance and anchored concordance (central)—to provide a more stable comparison of relative ordering across subsets. Figure 3 summarizes these effects across models, while full per-model results are provided in Table E.1 of Appendix E.

#### Events supported by structured EHR evidence.

Events with certain_EHR=1 exhibit consistently stronger temporal performance across models. As shown in Figure 3, AULTC improves uniformly across all models, and concordance increases for most models (five of seven). Anchored concordance metrics show similar patterns, with improved ordering relative to both the full event set and central anchors for a majority of models. While event match rate increases modestly, the primary gains are in temporal metrics. These results indicate that structured EHR rows serve as effective temporal calibrators when aligned with text-derived events, improving both absolute timestamp accuracy and relative ordering in many cases.

#### Model-reported temporal certainty.

In contrast, the certain flag does not reliably correspond to improved temporal quality. As illustrated in Figure 3, concordance and anchored concordance frequently decrease for certain=1 events compared to certain=0 events, even as AULTC increases across most models. Although events marked as certain often achieve higher event match rates, temporal ordering metrics do not consistently improve. This suggests that model-assigned confidence is more closely aligned with absolute timestamp placement than with preserving coherent temporal ordering, and therefore should be interpreted cautiously as a proxy for temporal reliability.

#### Central events as temporal anchors.

Central events exhibit a markedly different pattern. As shown in Figure 3, events with is_central=1 consistently yield substantial improvements in concordance, anchored concordance, and anchored concordance (central) across all models, indicating significantly stronger temporal ordering. These gains are consistent across both pairwise and anchor-relative evaluations, suggesting that central events are more robustly integrated into the global timeline structure. However, improvements in ordering are not always accompanied by gains in AULTC; in several models, absolute timestamp accuracy decreases for central events. This trade-off reflects the pipeline’s internal behavior: enforcing the strict relative sequence of the central scaffold comes at the expense of precise absolute temporal localization that is observed with slightly lower AULTC.

### 4.4. Information Missing from Tabular Data

We performed this auxiliary gap analysis using the timelines generated by GLM5, since it achieved the best temporal performance with the highest concordance and AULTC (Table 1). This provides the strongest available text-derived temporal view for assessing what clinically meaningful information remains absent, delayed, or compressed in structured tabular data. Detailed analyses are provided in Appendix C.

#### Structured coverage is incomplete.

Across 19 i2m4 cases and 2,756 textual events, only 983 events (35.7%) were well captured by tabular data, whereas 960 events (34.8%) had no structured counterpart at all. The remaining events exhibited partial but imperfect correspondence, including 422 temporal mismatches (15.3%), 307 semantically distant matches (11.1%), and 84 detail gaps (3.0%). Thus, structured data captures only part of the patient trajectory described in narrative text.

#### Timing is useful when tabular counterparts exist.

When a structured counterpart is present, its timing is often clinically useful. Among matched events, the median discrepancy between text and tabular timestamps was 2.6 hours; 64.4% of matched events fell within 6 hours, 74.7% within 12 hours, and 90.1% within 24 hours. This supports the central intuition of our multimodal pipeline: structured records are often valuable as temporal anchors even though they do not provide a complete representation of the clinical narrative.

#### Missing events remain clinically important.

The information missing from tabular data is not limited to low-value narrative detail. Missing events frequently included presenting symptoms, symptom progression, functional status, time-critical temporal qualifiers, and patient-reported outcomes, all of which may contribute to risk stratification and early detection. The same pattern appears in the forecasting-relevance analysis: among 312 high-relevance events, only 51.6% were well captured, while 35.9% had coverage or timing issues, including 19.2% with complete absence and 16.7% with temporal mismatch. Taken together, these findings suggest that tabular data is often useful for timing when present, but narrative text remains essential for recovering clinically meaningful event content.

## 5. Discussion and Conclusion

Our main results suggest that the benefit of multimodal alignment lies primarily in temporal calibration rather than event discovery. Across models, adding retrieved structured EHR evidence has little effect on event match rate, but it more consistently improves temporal concordance and, especially, AULTC. This indicates that structured rows are most useful not for expanding the set of events recovered from narrative text, but for sharpening when those text-derived events occurred. Put another way, structured EHR data is most effective in our framework as a temporal calibrator for text-derived event trajectories rather than as a substitute for narrative event content. The contrast between DeepSeek V3.2 and GLM5 further underscores this point: stronger event recovery does not necessarily imply better temporal localization on the matched subset. In this sense, the empirical effect of multimodal alignment is best understood as shifting the trade-off frontier toward higher temporal quality rather than uniformly improving all aspects of timeline reconstruction.

The ablation results provide evidence that these gains depend not only on access to structured data, but also on how that data is introduced into the reconstruction process. The weakest performance of the single-step formulation suggests that timeline reconstruction benefits from being factorized around temporally informative central events rather than solved in one pass. For DeepSeek V3.2, the strongest temporal performance is achieved when structured evidence is used both to refine the central scaffold and to recalibrate the final assembled timeline. Appendix results for DeepSeek R1 and Qwen3.5–397B generalize the first part of this conclusion: the multistep scaffold is consistently helpful across models, even though the best stage for multimodal calibration is somewhat model-dependent. Taken together, these findings support our central design hypothesis that structured evidence is most effective when introduced within a staged scaffolded pipeline, where it can first stabilize the temporal backbone of the encounter and then refine the expanded trajectory.

The sensitivity analyses further clarify when multimodal alignment helps most. Events supported by structured EHR evidence consistently achieve better absolute timestamp accuracy and often better temporal ordering, reinforcing the interpretation of tabular data as an external temporal anchor. Central events show a different pattern: they yield large gains in concordance and anchored concordance, indicating that the central scaffold improves global temporal structure, but these ordering gains do not always translate into better absolute timestamp accuracy. This suggests that the multistep pipeline is particularly effective at enforcing coherent chronology, even when precise absolute localization remains difficult. By contrast, model-reported certainty is a weaker signal: it is more closely associated with absolute timestamp placement than with correct temporal ordering, and therefore should not be treated as a reliable proxy for overall temporal fidelity.

Our gap analysis helps explain why multimodal timeline reconstruction is necessary in the first place. Structured EHR data is not merely a noisier version of the narrative record; it is a different view of the patient trajectory. When structured counterparts exist, they often provide useful temporal anchors, which is consistent with the temporal gains we observe from multimodal refinement. However, a substantial fraction of clinically meaningful events are absent, delayed, or semantically compressed in tabular form, including symptoms, progression, severity, and causal context. This means that text and tabular data should not be treated as interchangeable modalities. Rather, the results support the design choice underlying our pipeline: narrative text serves as the primary source of event content, while structured records provide partial but valuable evidence for improving temporal precision. More broadly, these findings suggest that models built only on structured data may miss clinically important signals even when their timestamps are precise, whereas models built only on text may recover those signals but place them less accurately in time.

### Limitations and future directions.

This study has some important limitations to consider. First, our evaluation is based on a relatively small set of manually annotated discharge summaries drawn from MIMIC-style critical care records. Although this benchmark is valuable for detailed temporal analysis, it reflects the documentation practices of a specific clinical research cohort and may not capture the full diversity of note structure, care settings, or disease presentations encountered in broader hospital systems. Second, the manual reference annotations are taken from the publicly available timeline resource of Frattallone-Llado et al. (2024), which was originally developed in an i2b2-style concept-centric format rather than directly for textual time-series. While we reformat these annotations to better match our TTS formulation, some representational mismatch likely remains and may contribute to lower event match rates, especially when compared with more directly aligned text-timeline settings. Third, the current benchmark remains limited in scale, constraining statistical power, limiting robustness analysis across event types and note styles, and preventing formal significance testing. Nevertheless, the consistent directional trends across all seven models suggest that the findings are not model-specific. Finally, our multistep graph design depends critically on the quality of central-event extraction. Because later stages are conditioned on these anchor events, errors in central-event selection or timing can propagate through the remainder of the pipeline and affect the final reconstructed timeline.

Several directions could extend this work. An immediate next step is to scale manual annotations beyond the current benchmark; ongoing efforts to expand annotation to a much larger set of discharge summaries will make it possible to evaluate temporal reconstruction more robustly and across a broader range of clinical cases. It will also be important to study generalization beyond sepsis, including which aspects of the multistep multimodal framework transfer naturally to other conditions and which depend on disease-specific documentation patterns or structured correlates. Another key question is how the framework should adapt when multimodal data is unavailable. Our results suggest that the central-anchor decomposition may still be useful in text-only settings, but this requires direct evaluation. Finally, a major downstream direction is to use reconstructed patient trajectories in predictive and causal modeling. Better-quality timelines may enable more faithful forecasting, treatment-response analysis, and trajectory-based decision support than models trained directly on retrospective narratives or coarse structured summaries.

## Figures and Tables

**Table 1:** Performance at event-matching threshold 0.1 for unimodal and multimodal timeline reconstruction. Bold indicates the best value within each metric-modality column.

Model	Event match rate		Concordance		AULTC	

	Unimodal	Multimodal	Unimodal	Multimodal	Unimodal	Multimodal

DeepSeek R1	0.501	0.502	0.784	0.788	0.817	0.820
DeepSeek V3.2	**0.588**	**0.588**	0.772	0.783	0.814	0.821
GLM5	0.319	0.319	**0.797**	**0.812**	**0.819**	**0.829**
KimiK2-Instruct	0.381	0.384	0.743	0.758	0.768	0.770
Qwen3.5-397B	0.580	0.580	0.776	0.759	0.802	0.809
GPT-OSS-120B	0.502	0.501	0.773	0.752	0.798	0.801
Mistral-4-Small	0.278	0.278	0.622	0.629	0.721	0.728

**Table 2:** Ablation results for DeepSeek V3.2. Bold indicates the best value in each metric-setting column. The “update central timeline only” condition yields a single final timeline and is therefore undefined for the unimodal setting.

Ablation	Event match rate		Concordance		AULTC	

	Unimodal	Multimodal	Unimodal	Multimodal	Unimodal	Multimodal

Single-step	0.420	0.420	0.758	0.781	0.809	0.819
Update central timeline only	–	0.548	–	0.749	–	0.817
Update final timeline only	**0.606**	**0.608**	0.739	0.731	0.805	0.809
Update both central and final timeline	0.588	0.588	**0.772**	**0.783**	**0.814**	**0.821**

**Figure E.1:**
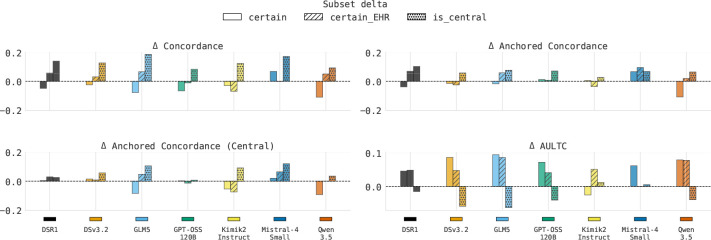
Sensitivity analysis across event subsets using the original manual annotations v1. Across all events, the proportion of flagged events is 79.0% for certain=1, 21.9% for certain_EHR=1, and 17.6% for is_central=1.

**Figure E.2:**
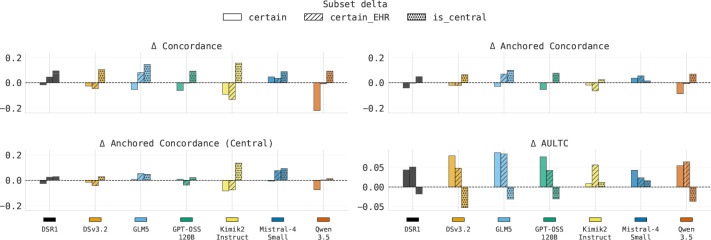
Sensitivity analysis across event subsets using rule-based cleaned annotations v2. Across all events, the proportion of flagged events is 78.1% for certain=1, 22.1% for certain_EHR=1, and 18.1% for is_central=1.

**Figure F.1:**
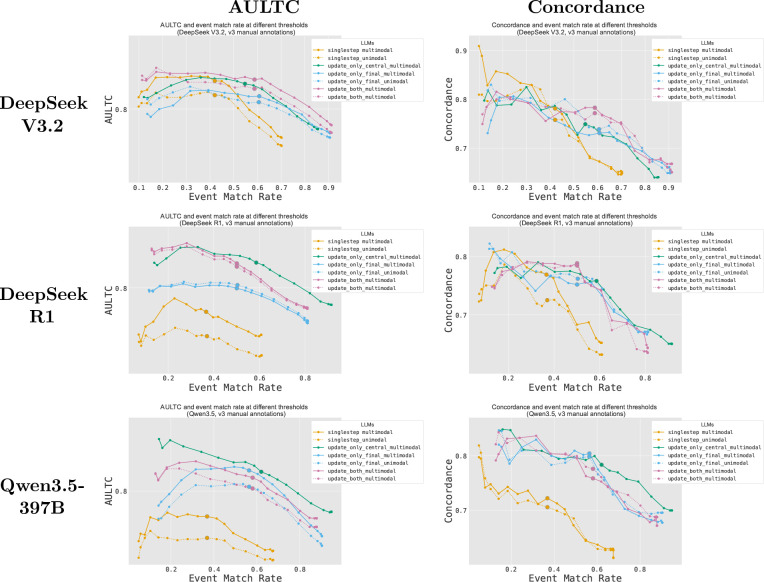
Threshold-sweep analyses for the three ablation models under (v3) manual annotations. Rows correspond to models and columns correspond to temporal metrics. In each panel, the event-matching threshold is varied from 0.01 to 0.50 in increments of 0.01, and the resulting event match rate is plotted against either AULTC or temporal concordance for the ablation variants.

**Figure G.2:**
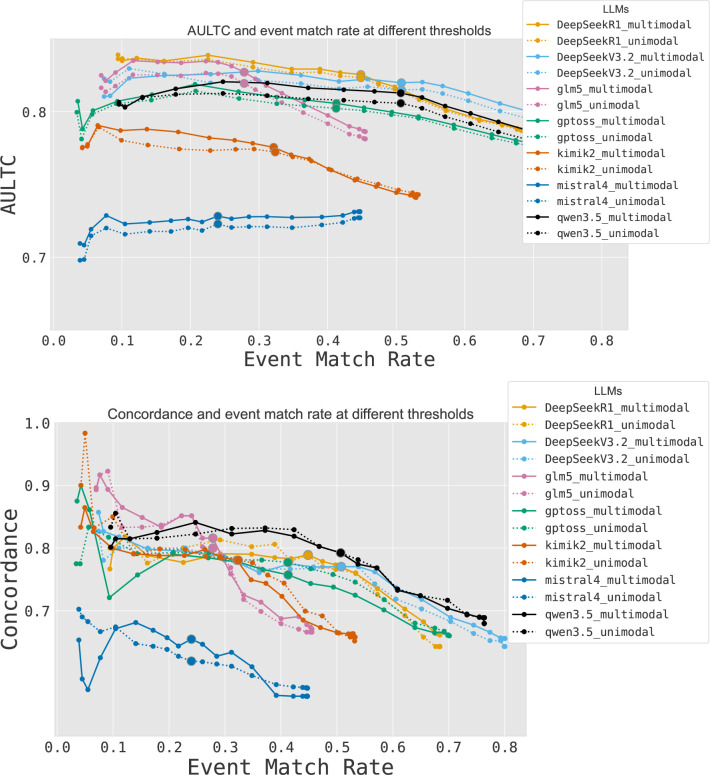
Temporal performance versus event match rate across event-matching thresholds for unimodal and multimodal variants of each model (v2 **version of manual gold standard annotations**). The top panel shows AULTC versus event match rate, and the bottom panel shows temporal concordance versus event match rate.

**Figure G.3:**
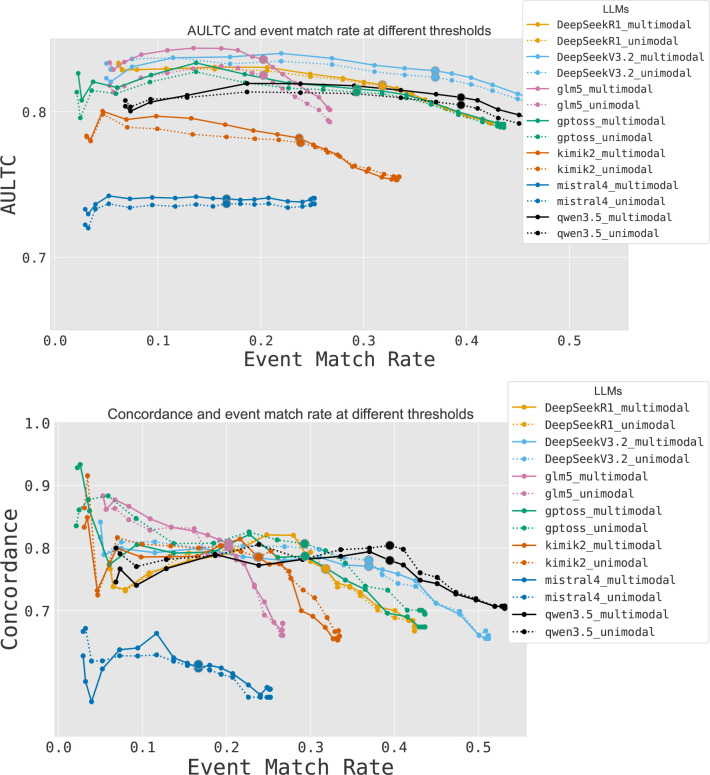
Temporal performance versus event match rate across event-matching thresholds for unimodal and multimodal variants of each model (v1 **version of manual gold standard annotations**). The top panel shows AULTC versus event match rate, and the bottom panel shows temporal concordance versus event match rate.

## Appendix

This appendix provides additional implementation details, prompt specifications, and extended empirical analyses that complement the main text. We organize the appendix into several components corresponding to different stages of the pipeline and evaluation.

### LLM annotation prompts.

Appendix A contains the full set of prompts used in our framework. These include prompts for extracting central events, estimating pairwise temporal relations, reconstructing the central scaffold, extracting non-central events, and reconstructing the full timeline. We also include prompts for multimodal timeline updates using structured EHR data, as well as a single-step unimodal baseline for comparison.

### Evaluation of textual time-series.

Appendix B provides a detailed description of the evaluation framework used throughout the paper. This includes definitions of event matching, temporal concordance, and AULTC, along with implementation details to ensure reproducibility and consistency across experiments.

### Gap analysis.

Appendix C analyzes information that is present in narrative text but missing from structured tabular data. This section formalizes the notion of gaps and describes how they are identified and categorized in our setting.

### Gold standard reformatting.

Appendix D describes the procedure used to reformat the manual annotations into a textual time-series representation compatible with our evaluation pipeline.

### Sensitivity analyses.

Appendix E reports extended sensitivity analyses, including stratified evaluations across subsets of events. These results provide a more granular understanding of model behavior under different conditions.

### Extended results for ablation variants.

Appendix F reports evaluation results for different variants of the default multimodal multistep pipeline. This includes variants that calibrate the central timeline only, the final timeline only, both stages, or neither stage, thereby isolating how retrieved structured evidence contributes to timeline quality.

### Extended results for multistep performance metrics.

Finally, Appendix G presents evaluation results at different event matching thresholds and across different versions of the reformatted gold standard. This provides a more detailed view of the trade-offs between event match rate and temporal accuracy.

Overall, this appendix is intended to provide sufficient detail to facilitate reproducibility of our pipeline and to support a deeper interpretation of the empirical findings presented in the main text. We will release our code on GitHub upon publication.

## Appendix A. LLM annotation prompts

### A.1. Prompt to extract central events from i2m4 discharge summaries

Extract central events Task: Extract all central (referent) events from the discharge summary. Events are findings or mentions about the individual that could involve or affect the health of the patient and that are temporally located. Central events are key to the patient’s timeline including events that other events are temporally referenced to. Guidelines:

- Use the original text span except for application of the contextual phrases such as perception time, ”history of” when needed for the event to stand alone.
- Separate conjunctive phrases into component events (e.g., ”fever and rash” or ”fever, rash” should be two events: ”fever”, ”rash”)
- Contextual phrases may be reapplied across component events (e.g. ”new onset of fever and rash” becomes ”new onset of fever” and ”new onset of rash”)
- For events with duration, use the start of the time interval as the event time.
- Output must be in BSV (Bar-Separated Values) format with a single header line.
- Only include the BSV data - no additional text or explanations

Example input:
An 18-year-old male was admitted to the hospital with a 3-day history of fever and rash. Four weeks ago, he was diagnosed with acne and received subsequent treatment with minocycline, 100 mg daily, for 3 weeks. With increased WBC count, eosinophilia, and systemic involvement, this patient was diagnosed with DRESS syndrome. The fever and rash persisted through admission, and diffuse erythematous or maculopapular eruption with pruritus was present. One day later the patient was discharged, and the rash resolved in another two days.
Example BSV output event

admitted to the hospital
diagnosed with acne
discharged
Output Instructions:

1. First line must be the header: ”event”.
2. Each subsequent line contains one central event.
3. No empty lines.
4. No additional text or explanations.
5. Events should be in chronological order when possible

### A.2. Prompt to compute time difference between pairs of central events

Pairwise temporal relations among central events Task: Compute time distances between pairs of central events. For each pair, provide:

- The two events (event1, event2).
- The time difference ($e_{2}\;-\;e_{1}$; event2 time - event1 time) in hours.
- A confidence score (1–9) in the certainty of this timing

Guidelines:

- Admission event (if present) is at time zero.
- If no admission event, use case presentation time as time zero.
- Events before time zero have negative timestamps.
- Events after time zero have positive timestamps.
- Use your expert clinical judgment to approximate timing when not explicitly stated.
- If exact timing cannot be determined, estimate a reasonable range.
- Never return null/undefined for $e_{2}\;-\;e_{1}$ - always provide a numeric estimate.
- Confidence scores:
  - 1–3: Low confidence (based only on indirect evidence).
  - 4–6: Moderate confidence (some direct evidence available).
  - 7–9: High confidence (explicit timing documentation).
- Low and medium confidence pairs may be omitted so long as each event has another pairing.
- Only include central event pairs that have a mention indicating a direct temporal relation.

Example input:
An 18-year-old male was admitted to the hospital with a 3-day history of fever and rash. Four weeks ago, he was diagnosed with acne and received subsequent treatment with minocycline, 100 mg daily, for 3 weeks. With increased WBC count, eosinophilia, and systemic involvement, this patient was diagnosed with DRESS syndrome. The fever and rash persisted through admission, and diffuse erythematous or maculopapular eruption with pruritus was present. One day later the patient was discharged, and the rash resolved in another two days.
Central events event

admitted to the hospital
diagnosed with acne
discharged
Example BSV Output event1 | event2 | $e_{2}\;-\;e_{1}$ | confidence

admitted to the hospital | diagnosed with acne | −672 | 9
discharged | admitted to the hospital | −24 | 9
Output Instructions:

1. Output must be in BSV (Bar-Separated Values) format
2. First line must be the header: ”event1 | event2 | $e_{2}\;-\;e_{1}$ | confidence”
3. Each subsequent line contains one event pair
4. No empty lines
5. No additional text or explanations

Required Fields:

- event1: First event in pair
- event2: Second event in pair
- $e_{2}\;-\;e_{1}$: Numeric value (event2 time - event1 time) in hours
- confidence: Integer between 1–9

### A.3. Prompt to extract central event timeline using central events and pairwise distances

Initial central timeline reconstruction You are a medical timeline reconstruction expert. Given a list of central events and their time distances, reconstruct the most likely and most precise timeline. Instructions:

1. Analyze all time distances to determine the most likely temporal order.
2. Assign time 0 to the time of admission, if available, or else to the time of case presentation.
3. For each subsequent event, calculate its time based on the time distances.
4. When there are conflicting time distances, use the one with higher confidence.
5. Output the timeline in BSV format with headers event | time

Example input:
An 18-year-old male was admitted to the hospital with a 3-day history of fever and rash. Four weeks ago, he was diagnosed with acne and received subsequent treatment with minocycline, 100 mg daily, for 3 weeks. With increased WBC count, eosinophilia, and systemic involvement, this patient was diagnosed with DRESS syndrome. The fever and rash persisted through admission, and diffuse erythematous or maculopapular eruption with pruritus was present. One day later the patient was discharged, and the rash resolved in another two days.
Central events event

admitted to the hospital
diagnosed with acne
discharged
Example central time distances (event2 - event1): event1 | event2 | $e_{2}\;-\;e_{1}$ | confidence

admitted to the hospital | diagnosed with acne | −672 | 9
discharged | admitted to the hospital | −24 | 9
Example output: event | time admitted to the hospital | 0

diagnosed with acne | −672
discharged | 24
Output format: Output must be in this exact BSV format: event | time event1 | time1 event2 | time2

### A.4. Prompt to extract non-central events from i2m4 discharge summaries and their timing with respect to central events

Extract non-central events Task: Extract clinical events from text. Events are findings or mentions **about the individual** that could involve or affect the health of the patient and that are temporally located. For each event, provide:

- The event
- A referent central event (from the provided list), which is an event that serves as a temporal reference.
- Time difference in hours (negative for before, positive for after the central event).
- A confidence score about the time difference (0: low, 9: high).

Guidelines:

1. Include all events except those listed in ’central events’, even if in discussion.
2. Do not omit any events. Include termination and discontinuation events.
3. Include pertinent negative findings (e.g., ”no shortness of breath”).
4. Separate conjunctive phrases into component events (e.g., ”fever and rash” or ”fever, rash” becomes ”fever”, ”rash”).
5. Contextual phrases may be reapplied across component events (e.g. ”new onset of fever and rash” becomes ”new onset of fever” and ”new onset of rash”).
6. For events with duration, use the start of the time interval as the event time.
7. Use your expert clinical judgment to approximate timing when not explicitly stated.
8. Output must be in BSV (Bar-Separated Values) format.
9. No additional text or explanations - only the BSV data

Example input:
An 18-year-old male was admitted to the hospital with a 3-day history of fever and rash. Four weeks ago, he was diagnosed with acne and received subsequent treatment with minocycline, 100 mg daily, for 3 weeks. With increased WBC count, eosinophilia, and systemic involvement, this patient was diagnosed with DRESS syndrome. The fever and rash persisted through admission, and diffuse erythematous or maculopapular eruption with pruritus was present. One day later the patient was discharged, and the rash resolved in another two days.
Central events:
admitted to the hospital
diagnosed with acne
discharge
Example BSV output: event | central event | relative time | confidence

18 years old|admitted to the hospital|0|9
male|admitted to the hospital|0|9
fever|admitted to the hospital|−72|8
rash|admitted to the hospital|−72|8
treatment with minocycline|diagnosed with acne|0|7
increased WBC count|admitted to the hospital|0|5
eosinophilia|admitted to the hospital|0|5
systemic involvement|admitted to the hospital|0|5
diffuse erythematous or maculopapular eruption|admitted to the hospital|0|5
pruritus|admitted to the hospital|0|5
DRESS syndrome|admitted to the hospital|0|5
fever persisted|admitted to the hospital|0|7
rash persisted|admitted to the hospital|0|7
rash resolved|discharge|48|9
Output instructions:

1. First line must be the header: event | central event | relative time | confidence.
2. Each subsequent line contains one event with its temporal reference.
3. No empty lines.
4. No additional text or explanations.
5. All fields must be present for each row.
6. relative time must be numeric (can be negative).
7. confidence must be integer between 0–9.

Required Fields:

- event: The non-central event text.
- central event: The reference central event.
- relative time: Hours difference from central event (negative before, positive after).
- confidence: Certainty score (0–9).

### A.5. Prompt to reconstruct full timeline (central + non-central)

Reconstruct full timeline Task: Reconstruct the complete timeline with absolute times.

- Admission event (if present) is at time zero.
- If no admission event, use case presentation time as time zero.
- For central events: use the provided central event pairing time distances with preference for pairing with high confidence and resolve as necessary using the context from the discharge summary.
- For non-central events: calculate absolute time by adding relative time to mapped central event’s absolute time and resolve as necessary using the context from the discharge summary.
- Events before time zero have negative timestamps.
- Events after time zero have positive timestamps

Guidelines:

1. Use hours as the time unit.
2. Omit the unit from output (implied hours).
3. For events with duration, use the start of the time interval.
4. Include **all** events (both central and non-central events).
5. Cross-reference with original discharge summary for accurate timing.
6. Output must be in BSV (Bar-Separated Values) format.
7. No additional text or explanations - only the BSV data.

Example input:
An 18-year-old male was admitted to the hospital with a 3-day history of fever and rash. Four weeks ago, he was diagnosed with acne and received subsequent treatment with minocycline, 100 mg daily, for 3 weeks. With increased WBC count, eosinophilia, and systemic involvement, this patient was diagnosed with DRESS syndrome. The fever and rash persisted through admission, and diffuse erythematous or maculopapular eruption with pruritus was present. One day later the patient was discharged, and the rash resolved in another two days.
Example central timeline file: event — time

admitted to the hospital | 0
diagnosed with acne | −672
discharge | 24
Example non-central events file: event | central event | relative time | confidence

18 years old|admitted to the hospital|0|9
male|admitted to the hospital|0|9
fever|admitted to the hospital|−72|8
rash|admitted to the hospital|−72|8
treatment with minocycline|diagnosed with acne|0|7
increased WBC count|admitted to the hospital|0|5
eosinophilia|admitted to the hospital|0|5
systemic involvement|admitted to the hospital|0|5
diffuse erythematous or maculopapular eruption|admitted to the hospital|0|5
pruritus|admitted to the hospital|0|5
DRESS syndrome|admitted to the hospital|0|5
fever persisted|admitted to the hospital|0|7
rash persisted|admitted to the hospital|0|7
rash resolved|discharge|48|9
Example BSV output event | time

18 years old|0
male|0
admitted to the hospital|0
fever|−72
rash|−72
acne|−672
minocycline|−672
increased WBC count|0
eosinophilia|0
systemic involvement|0
diffuse erythematous or maculopapular eruption|0
pruritus|0
DRESS syndrome|0
fever persisted|0
rash persisted|0
discharged|24
rash resolved|72
Output instructions:

1. First line must be the header: event | time.
2. Each subsequent line contains one event with its absolute time.
3. No empty lines.
4. No additional text or explanations.
5. All fields must be present for each row.
6. Time must be numeric (can be negative)

Required Fields:

- event: The event description.
- time: Absolute time in hours (negative before time zero, positive after)

### A.6. Prompt to integrate information from structured EHR to update timeline (central and final)

Update timeline (central/final) with information from structured data Task: You are a medical timeline calibration expert. Your task is to **adjust the timing of clinical events** using the **top-10 nearest structured EHR rows** retrieved for each event. You will be provided with a discharge summary or note, a list of clinical events (with the initial timing), and the top-10 most similar structured EHR rows, or evidences, in BSV format for each event. Using these structured EHR rows, modify the event time only if the structured evidence justifies it. Instructions

- For each clinical event, analyze all the top-10 EHR rows. Use **only clinically relevant rows** to refine the event timing with your expert clinical judgment.
- If no rows are relevant, keep the initial timing unchanged.
- Each EHR row is provided in BSV format as name | value | time | similarity, where name is clinical feature/observation, value is measurement (NaN if unavailable), time is in hours relative to admission, and similarity is with the event in the embedding space.
- Rows that have the same name and value are merged, and their times are listed in the same cell. If a row with multiple timings is clinically relevant to the event, try choosing the most relevant time from the context found in the discharge summary.
- Assign time 0 to the time of admission, if available, or else to the time of case presentation.
- When there are conflicting rows, use the most relevant and confident one.
- Output the timeline in BSV format with headers event | time. Write only one row for each event.

Example input:
An 18-year-old male was admitted to the hospital with a 3-day history of fever and rash. Four weeks ago, he was diagnosed with acne and received subsequent treatment with minocycline, 100 mg daily, for 3 weeks. With increased WBC count, eosinophilia, and systemic involvement, this patient was diagnosed with DRESS syndrome. The fever and rash persisted through admission, and diffuse erythematous or maculopapular eruption with pruritus was present. One day later the patient was discharged, and the rash resolved in another two days.
Example events with top-10 Rows: Event 1: admitted to the hospital, time: 0 Top-10 EHR rows: name | value | time | similarity

admission type:emergency | nan | 0.0 | 0.774
admission location:emergency room admit | nan | 0.0 | 0.753
chart:temperature | 38.7 | 4.47 10.47 13.47 | 0.701
chart:heart rhythm: | Normal Sinus | 1.47 3.47 11.47 16.47 | 0.654
chart:hematocrit: | 34 | 0.5 | 0.612
lab:blood:hematology:d-dimer | 220 | 1.0 | 0.602
chart:platelets: | 210 | 0.5 | 0.595
chart:chest tube site: | Left Anterior | 0.7 | 0.581
chart:respiratory rate:bpm | 22 | 0.3 | 0.556
chart:spo2 | 92 | 0.2 | 0.541
Event 2: diagnosed with acne, time: −672 Top-10 EHR rows: name | value | time | similarity

codx:acne vulgaris | nan | −672.0 | 0.789
chart:skin condition: | Papulopustular | −672.0 | 0.742
chart:medication order:minocycline | 100 mg PO daily | −672.0 | 0.721
lab:blood:chemistry:alanine aminotransferase | 18 | −671.0 | 0.615
chart:allergy | None | −672.0 | 0.604
chart:follow up service:dermatology | Scheduled | −648.0 | 0.588
chart:skin integrity: | Intact | −672.0 | 0.562
chart:braden mobility: | Independent | −672.0 | 0.545
chart:service: | TRA | −672.0 | 0.532
chart:readmission: | nan | −672.0 | 0.518
Event 3: discharged, time: 24.0 Top-10 EHR rows: name | value | time | similarity

discharge location:home | nan | 26.98 | 0.663
chart:iv [site]: | nan | 25.0 | 0.623
chart:hematocrit: | 36 | 25.0 | 0.601
chart:hemoglobin:gm/dl | 12.2 | 25.0 | 0.588
chart:skin integrity: | Improving | 25.0 | 0.577
chart:respiratory rate:bpm | 18 | 25.0 | 0.559
chart:pain level: | 2-Mild | 25.0 | 0.541
chart:follow up | Primary care | 26.98 | 0.528
chart:removed x 5 mins: | Done | 26.98 | 0.511
chart:readmission: | nan | −672.0 | 0.480
Example output: event | time

admitted to the hospital | 0
diagnosed with acne | −672
discharged | 26.98
Output format: Output must be in this exact BSV format: event | time event1 | time1 event2 | time2

### A.7. Prompt to extract timeline for all events in a single-step workflow

Singestep timeline extraction Task: You are a physician. Extract clinical events and their timestamps (in hours) from the discharge summary below. Definitions and rules:

- Use the admission event as timestamp 0.
- If an explicit admission event is not stated, choose the main presenting problem/diagnosis/treatment at the start of the hospitalization as timestamp 0.
- Events that occurred before timestamp 0 must have negative timestamps. Events after must have positive timestamps.
- Timestamps must be numeric values in hours. Do NOT include units.
- If a time is not explicitly stated, approximate it using temporal expressions in the text and clinical reasoning. Use the start time for events with duration.
- Separate conjunctive phrases into individual events and assign them the same timestamp (e.g., fever and rash → fever, rash).
- Use the original text span as the event whenever possible (minimal normalization; remove only leading phrases like ”history of” where appropriate).
- Include all patient-related events mentioned anywhere in the summary, including:
  - diagnoses, symptoms, signs, labs, imaging, procedures, medications, interventions.
  - discontinuation/termination events (e.g., ”stopped X”).
  - pertinent negatives (e.g., ”no shortness of breath”, ”denies chest pain”).
- Add a confidence score (1–9) in the certainty of this timing. This confidence score reflects how certain you are that the timestamp was directly derived from the text, as opposed to being approximated using clinical judgment when explicit temporal information was not available.
- Confidence scores:
  - 1–3: Low confidence (based only on indirect evidence).
  - 4–6: Moderate confidence (some direct evidence available).
  - 7–9: High confidence (explicit timing documentation).

Example input:
An 18-year-old male was admitted to the hospital with a 3-day history of fever and rash. Four weeks ago, he was diagnosed with acne and received subsequent treatment with minocycline, 100 mg daily, for 3 weeks. With increased WBC count, eosinophilia, and systemic involvement, this patient was diagnosed with DRESS syndrome. The fever and rash persisted through admission, and diffuse erythematous or maculopapular eruption with pruritus was present. One day later the patient was discharged, and the rash resolved in another two days.
Example BSV output event | time | confidence

18 years old|0|9
male|0|9
admitted to the hospital|0|9
fever|−72|8
rash|−72|8
acne|−672|8
treatment with minocycline|−672|7
increased WBC count|0|5
eosinophilia|0|5
systemic involvement|0|5
diffuse erythematous or maculopapular eruption|0|5
pruritus|0|5
DRESS syndrome|0|5
fever persisted|0|7
rash persisted|0|7
discharged|24|9
rash resolved|72|9
Output format requirements (STRICT):

1. Return only a raw bar-separated table and nothing else.
2. The first line must be exactly the header: event | time | confidence.
3. Each following line must contain:event | time | confidence..
4. Output ONLY the table. No extra text. No bullet points. No Markdown/code fences. No blank lines. No explanation.
5. Use numeric time values only and use numeric confidence values only.
6. Do not include markdown, bullets, code fences, or explanatory text.

## Appendix B. Evaluation of textual time-series

We evaluated textual time series derived from PMOA case reports along three complementary axes: (i) semantic correspondence between predicted events and manually annotated events (event match rate), (ii) consistency in temporal ordering (temporal concordance), and (iii) similarity in timestamp values (time discrepancy). Together, these metrics capture different aspects of timeline quality.

### B.1. Event Match Rate

To quantify how well predicted clinical events correspond to reference events, we adopt a recursive best-match procedure adapted from Wang and Weiss (2025); Noroozizadeh and Weiss (2026), as illustrated in Algorithm B.1. At each iteration, the procedure selects the closest unmatched pair of predicted and reference events according to a text-similarity metric, retains the pair if it meets a distance threshold, and then removes both events before continuing. **Algorithm B.1** presents pseudocode for this recursive matching procedure. This approach yields a one-to-one alignment between reference and predicted events and is efficient for timelines of unequal length.

We compared multiple similarity measures, including Levenshtein distance, BERT-based embeddings, and PubMedBERT embeddings, and found that cosine similarity computed over PubMedBERT sentence embeddings gave the best performance. A cosine distance threshold of 0.1 is used to decide whether a predicted event qualifies as a semantic match.

Under this procedure, the event match rate is defined as:

$$\text{Match}\;\text{Rate}=\frac{\#\{\text{reference}\;\text{events}\;\text{with}\;\text{a}\;\text{matched}\;\text{prediction}\}}{\#\{\text{reference}\;\text{events}\}},$$

which represents the proportion of reference events that are successfully recovered.

### B.2. Temporal Concordance

We measure temporal ordering accuracy using the concordance index (c-index), which quantifies the probability that matched event pairs appear in the correct relative order in predicted time. Let $t_{i}^{\text{ref}}$ and $t_{i}^{\text{pred}}$ denote the reference and predicted timestamps for matched event i. The c-index is:

$$\text{c-index}=\frac{1}{N}\sum_{\begin{array}{c} i<j\\ t_{i}^{\text{ref}}\neq t_{j}^{\text{ref}}\\ t_{i}^{\text{red}}\neq t_{j}^{\text{pred}} \end{array}}1\left\{(t_{i}^{\text{ref}}-t_{j}^{\text{ref}})(t_{i}^{\text{pred}}-t_{j}^{\text{pred}})>0\right\},$$

where N is the number of comparable pairs. Higher values indicate better preservation of the reference ordering.

### B.3. Time Discrepancy and AULTC

Following the procedure in Noroozizadeh and Weiss (2026), for each matched event, timestamp accuracy is measured using the absolute time error $\Delta t_{i}=|t_{i}^{\text{pred}}-t_{i}^{\text{ref}}|$. Because these discrepancies may span several orders of magnitude, we analyze them on a log scale:

$$x_{i}=log\left(1+\Delta t_{i}\right).$$

To summarize timestamp accuracy across the dataset, we compute the empirical CDF over log-time discrepancies pooled across all matched events in the 20-case gold standard:

$$F(x)=\frac{1}{k}\sum_{i=1}^{k}1\left\{x_{i}\leq x\right\},$$

where k is the total number of matched events across all annotated cases.

We then summarize overall discrepancy using the Area Under the Log-Time CDF (AULTC):

$$AULTC=\frac{1}{log\left(1\;+\;S_{max}\right)}\left[\sum_{i=1}^{k}\left(x_{\left(i\right)}-x_{\left(i-1\right)}\right)\frac{i}{k}+\left(log\left(1+S_{max}\right)-x_{\left(k\right)}\right)\right],$$

where $x_{\left(i\right)}$ are the sorted log discrepancies and $S_{\text{max}}$ is the maximum observed absolute error. AULTC ranges from 0 to 1, with larger values indicating closer agreement between predicted and reference timestamps.

Finally, we stratify timestamp errors by temporal distance (e.g., within 1 hour, 1 day, 1 week, 1 year) to assess how accuracy changes across clinically meaningful time scales.

## Appendix C. Information Missing from Tabular Data

To better understand why multimodal timeline reconstruction is needed, we performed an auxiliary gap analysis comparing text-derived clinical events against structured EHR counterparts for the same encounters. Unless otherwise stated, this analysis uses the timelines generated by GLM5, which achieved the strongest temporal performance in the main evaluation. The purpose of this analysis is not to evaluate timeline reconstruction itself, but to characterize which clinically relevant aspects of the patient trajectory are preserved, delayed, compressed, or absent in structured tabular data.

### C.1. Gap-detection methodology

The analysis was performed on i2m4 cases with complete aligned text and structured data (19 of 20 cases), using hospital admission time as the shared temporal anchor (t=0). The textual view consisted of event–time pairs from the best temporally performing LLM-derived timelines. The structured view consisted of the aligned tabular EHR record, including laboratory values, physiologic measurements, diagnoses, medications, and other structured rows associated with the same encounter.

For each textual event, we searched for a candidate tabular counterpart using embedding-based similarity matching. Candidate matches were then evaluated along two dimensions: semantic adequacy and temporal alignment. Semantic adequacy was assessed using retrieval-augmented scoring on a 0–1 scale, reflecting whether the structured record captured the same clinical content as the textual event. Temporal alignment was measured as the absolute difference in hours between the text-derived timestamp and the matched tabular timestamp.

Based on these criteria, each textual event was assigned to one of five categories: *well captured*, *complete absence*, *temporal mismatch*, *semantic distance*, or *detail gap*. A textual event was considered *well captured* when a tabular match existed, the temporal difference was ≤ 12 hours, and the semantic score was ≥ 0.6. *Complete absence* indicates that no tabular match was found. *Temporal mismatch* indicates that a match existed but differed by more than 12 hours. *Semantic distance* indicates that a match existed but had semantic score < 0.6. *Detail gap* indicates that a match existed but clinically important attributes like severity, progression, laterality, or duration were missing from structured representation.

### C.2. Overall summary of gap categories

Table C.1 summarizes the overall distribution of gap categories. Across 19 i2m4 cases and 2,756 textual events, only 983 events (35.7%) were well captured by tabular data, whereas 960 events (34.8%) had no structured counterpart at all. The remaining events exhibited partial but imperfect correspondence, including 422 temporal mismatches (15.3%), 307 semantically distant matches (11.1%), and 84 detail gaps (3.0%). These results show that structured data captures only part of the patient trajectory described in narrative text. At the same time, when a structured counterpart does exist, its timing is often clinically useful: among matched events, the median temporal alignment was 2.6 hours, with 64.4% within 6 hours, 74.7% within 12 hours, and 90.1% within 24 hours.

**Table C.1:** Summary of gap categories in the tabular-gap analysis. Percentages are computed over 2,756 textual events from 19 i2m4 cases. Temporal alignment statistics are reported only for events with tabular counterparts.

Metric	Value

Total cases	19
Total textual events	2,756
Well captured	983 (35.7%)
Complete absence	960 (34.8%)
Temporal mismatch	422 (15.3%)
Semantic distance	307 (11.1%)
Detail gap	84 (3.0%)

Median temporal alignment	2.6 h
Within 6 h	64.4%
Within 12 h	74.7%
Within 24 h	90.1%

**Table C.2:** Content categories among complete-absence events. Percentages are computed over the 960 textual events with no tabular counterpart. Categories are not mutually exclusive.

Category	Count	%	Example

Denial statements	170	17.7%	“no seizure-like activity”, “no deformity”
Symptom descriptions	52	5.4%	“chest pain”, “substernal chest pain for 1 day”
Temporal patterns	44	4.6%	“pain resolved”, “improving clots”
Severity quantification	40	4.2%	“stage IIIA”, “EF 55%”, “moderate defect”
Resolution status	31	3.2%	“resolved”, “improved”, “stable”
Causal factors	25	2.6%	“after surgery”, “complication”, “procedure”
Functional status	13	1.4%	mobility, activity, independence

### C.3. Further characterization of tabular gaps

To better understand what is missing from structured data, we manually grouped complete-absence events into clinically meaningful content categories. As shown in Table C.2, missing events frequently included denial statements, symptom descriptions, temporal patterns, severity quantification, resolution status, causal factors, and functional status. These categories are not mutually exclusive, and many events did not fit neatly into a predefined bucket, underscoring the diversity of clinically relevant information from narrative text.

Table C.3(a) shows the lag distribution for events whose tabular counterparts differed from text by more than 12 hours. The largest bin was 12–24 hours, but a substantial fraction extended beyond 72 hours, consistent with documentation lag, retrospective coding, and the fact that narrative text often mentions earlier symptoms or historical events before they appear in the structured record. We also stratified events by forecasting relevance. Table C.3(b) summarizes the overall relevance distribution, and Table C.3(c) shows that among 312 high-relevance events (11.3% of all events), only 51.6% were well captured, while 35.9% exhibited either complete absence or temporal mismatch. Thus, the missing information is not limited to peripheral narrative detail; it affects events most likely to matter for early warning, risk stratification, and trajectory modeling.

**Table C.3:** Further characterization of tabular gaps. (a) Distribution of temporal mismatch magnitudes for the 422 events whose tabular counterparts differed from text by more than 12 hours. (b) Distribution of events by forecasting relevance. (c) Gap distribution for high-relevance events.

(a) Temporal mismatch magnitudes		

Lag bin	Count	%

12–24 h	175	41.5%
24–48 h	98	23.2%
48–72 h	53	12.6%
72+ h	96	22.7%

(b) Event distribution by forecasting relevance		

Relevance	Count	%

High	312	11.3%
Medium	891	32.3%
Low	1,553	56.4%

(c) High-relevance gap distribution	

Metric	Value

High-relevance events	312 (11.3%)
Well captured	51.6%
Coverage/timing issues	35.9%
Complete absence	19.2%
Temporal mismatch	16.7%

Qualitatively, the gap taxonomy revealed several recurrent patterns. In some cases, tabular data captured downstream outcomes but not upstream causes, such as prior surgery or earlier symptom onset. In others, text recorded clinically important events hours before they became specific in structured data. Even when a tabular counterpart existed, it often compressed away severity, progression, triggers, or contextual interpretation, and some structured matches omitted modifiers such as laterality or duration that may materially affect clinical assessment.

#### Summary.

Taken together, this analysis shows that structured data is often useful for temporal anchoring when a counterpart exists, but it does not provide a complete account of the patient trajectory. Narrative text remains essential because it preserves symptom content, progression, context, and causal structure that are frequently absent, delayed, or compressed in tabular form. These findings provide additional motivation for the multimodal reconstruction framework developed in the main paper, in which text serves as the primary source of event content and structured EHR data serves as a complementary source of temporal evidence.

## Appendix D. Reformatting of gold standard annotations

### D.1. Reformatting procedure

The publicly released i2m4 timeline annotations provide expert event–time labels, but they were not originally constructed for the textual time series (TTS) formulation used in this work. In particular, the released annotations follow an i2b2-style, concept-centric representation that does not always align with our requirement that each event be a standalone, semantically interpretable patient finding. To make evaluation against LLM-generated TTS outputs as fair as possible, we therefore study three versions of the manual gold standard, corresponding to increasingly TTS-compatible levels of processing.

#### Version 1: original released annotations v1.

This version uses the annotations exactly as released by Frattallone-Llado et al. (2024), without any modifications, providing a direct baseline for evaluating performance against the original reference representation.

#### Version 2: rule-based preprocessing v2.

This version applies only minimal deterministic cleanup to v1. Specifically, we lowercase all event strings, normalize whitespace, remove exact duplicate event | time pairs, and delete section headers that do not satisfy our TTS definition of a standalone event. The removed headers include *admission*, *discharge*, *history of present illness*, *physical exam*, *pertinent results*, *brief hospital course*, *discharge medications*, *discharge diagnoses*, and *followup instructions*. This version isolates the effect of basic formatting and obvious non-event cleanup.

#### Version 3: LLM-based TTS reformatting v3.

Starting from v2, we apply a reasoning model to convert the cleaned annotations into a representation that more closely matches the TTS task. Importantly, the model operates only on the released annotation strings and their associated timestamps; it does *not* access the raw discharge summaries. The reformatting preserves the original physician-provided times while revising event strings to better satisfy the TTS definition. In particular, it splits conjunctive findings into separate events when appropriate, removes fragments that cannot be interpreted as standalone patient events, preserves clinically meaningful negatives, expands common abbreviations, and combines semantically related fragments when doing so yields a clearer standalone event. Thus, v3 addresses representational mismatch rather than re-annotating source documents.

Importantly, we acknowledge that reformatting the gold standard using an LLM could in principle create a circular evaluation if the reformatted reference systematically favors LLM-generated outputs; *however*, because the reformatting model operates solely on the released annotation strings and their timestamps—never on the raw discharge summaries—and because we manually verify that all v3 events remain grounded in the original physician-provided annotations, any stylistic alignment between v3 and LLM outputs reflects convergence toward the TTS representational target rather than a substantive advantage in temporal content.

Taken together, these three versions let us separate two distinct sources of evaluation mismatch. The comparison between v1 and v2 measures the effect of trivial normalization and removal of obvious non-events. The comparison between v2 and v3 measures the effect of deeper semantic alignment between the released concept-centric annotations and the standalone event representation required by TTS. We also provide the prompt (Llama 3.3 70B) used for the conversion v2 → v3.

Prompt to reformat manual annotations (v2 → v3) Task: You are a clinical expert reviewing manually annotated clinical events and converting them into Textual Time Series (TTS) format based solely on the event strings provided. You will not have access to the original case report. Your goal is to revise each event according to TTS annotation guidelines while preserving the time value. We define the term textual time series as a list of clinical findings each with an associated timestamp (which may be relative to time of case presentation) pertaining to an individual. A clinical finding is a free-text specification of an entity pertaining to or with the potential to affect the person’s health. Each input line has the format: event original | time Each output line must have the format: event original | event updated | time If an event is deleted, output: event original | | NA You must produce at least one output line for every input line. TTS annotation guideline

1. **Split and events**.If one event contains two clinical findings joined by “and”, output separate events. Example: “nausea and vomiting” - one line for “nausea”, one line for “vomiting”, both using the same time.
2. **Stand-alone events only**. An event updated must make sense by itself (outside the note). - Very vague fragments like ”color, ”laying”, ”this twin” are deleted unless combined into a meaningful event (see rule 7). - If it cannot be made stand-alone, output event original | | NA.
3. **Keep clinically meaningful negatives**. Keep events like “no shortness of breath”, “denies chest pain”, “afebrile”, if they clearly express the patient’s state.
4. **Remove duplicates**. If the same event (same wording and same time) appears more than once, keep only one output line for that concept.
5. **Expand common lab abbreviations** Expand lab abbreviations in event updated.
  - WBC → white blood cell count
  - Hgb → hemoglobin
  - Hct → hematocrit
  - Plt → platelet count
  - BUN → blood urea nitrogen
  - Cr / Creat → creatinine
  - Na → sodium
  - K → potassium
  - Mg → magnesium
    Example: “WBC 12.3” → “white blood cell count 12.3”.
6. **Combine fragments into a meaningful event when possible**. If short fragments **have the same timestamp** and clearly belong together semantically, combine them into one stand-alone event. Do not change the time. Examples:
  - ”transferred” + ”floor” → “transferred to floor”
  - ”fall” + ”bicycle” → fell off bicycle
  - Use the time of the more informative/primary event (usually the first event in the pair).

### D.2. Characterization of the Original-to-Preprocessed (v1 to v2) Annotation Transformation

The publicly released annotations follow an i2b2-style, concept-centric representation, where a single discharge summary is decomposed into many short and often context-dependent strings. Before using these annotations as an evaluation reference for LLM-generated textual time series, we apply a lightweight deterministic preprocessing step (v1→v2). This step includes lowercasing, whitespace normalization, removal of nine fixed section headers (e.g., *admission*, *discharge*, *history of present illness*), and deduplication of exact event,|,time pairs.

Table D.1 summarizes the effect of this preprocessing step across all 20 annotated cases.

**Table D.1:** Aggregate statistics of the original-to-preprocessed (v1 → v2) transformation across 20 annotated cases. *Other removed* denotes entries not matched to any v2 entry and not captured by the hardcoded header or duplicate rules.

	Count	% of v1

Total original v1 entries	3,785	100.0
Retained in preprocessed v2	2,169	57.3

Removed: known section headers	91	2.4
Removed: exact duplicates	111	2.9
Removed: other	1,555	41.1

The preprocessing retains 57.3% of v1 entries. The hardcoded header removal and deduplication rules together account for only 5.3% of v1, while the remaining 41.1% fall into an *other removed* category that is not directly explained by these rules.

#### Manual review of removed entries.

To better understand the *other removed* category, we randomly sampled up to 25 entries per case (455 entries in total across all 20 cases). Each entry was manually reviewed against both the v1 annotation string and the original discharge summary text, and then assigned to one of six mutually exclusive categories defined in Table D.2. Representative examples are provided in the table alongside each category.

#### Interpretation of the reformatting.

The results show that most entries in the *other removed* category do not correspond to meaningful clinical information. Standalone fragments (41.8%) and unlisted section or subsection headers (16.5%) together make up 58.3% of the sample and reflect artifacts of the i2b2 annotation style, where continuous text is broken into tokens and structural labels. An additional 9.5% correspond to physical exam or results table entries that do not function as standalone clinical findings. Entries labeled as expanded or renamed in v2 (19.3%) are not actually lost. Instead, they appear in v2 under a slightly different surface form, such as abbreviation expansion or minor text normalization, and are therefore not matched by exact string comparison above. Similarly, the 4.8% categorized as duplicates missed by the previous step do not represent loss of information. Only 8.1% of the sampled *other removed* entries correspond to genuine clinical findings that are absent from the preprocessed annotations. This amounts to approximately 3.3% of all v1 entries. Overall, these findings suggest that the v1→v2 transformation primarily removes representational noise introduced by the concept-centric i2b2 annotation scheme, rather than discarding clinically meaningful content. As a result, this preprocessed annotations provide a cleaner and more suitable reference for evaluating TTS outputs.

**Table D.2:** Manual review of 455 randomly sampled *other removed* entries (up to 25 per case), reviewed against the original discharge summary text. *Genuine clinical removal* is the only category representing a true loss of clinical information relative to the preprocessed reference.

Category	n	%	Example

Standalone fragment	190	41.8	Single words or short phrases with no standalone meaning outside the note: *radiating, born, well, ABDOMEN*, struck
Expanded/renamed in v2	88	19.3	Entry present in v2 under a semantically equivalent reformulation, most commonly abbreviation expansion (*BUN* → *blood urea nitrogen*) or removal of extraneous quotation marks (*bypass grafting”* → *bypass grafting*)
Unlisted section header	75	16.5	Structural note headers not covered by the nine hardcoded entries: *Chief Complaint, Major Surgical or Invasive Procedure, CT HEAD, IMPRESSION*
Physical exam / lab table entry	43	9.5	Rows of a structured physical exam or results table rather than free-text findings: *VS, HEENT, CTA, MMM, Neuro*
Genuine clinical removal	37	8.1	A real patient finding present in the original note with no counterpart in the preprocessed annotation: *afebrile, Regular rate, relatively stable*
Duplicate (missed by script)	22	4.8	Near-exact duplicate of another v1 entry not detected by the string-matching rule, typically due to quotation mark or punctuation variation: *rhythm”* vs. *rhythm*

Total	455	100.0	

### D.3. Characterization of the Preprocessed-to-Reformatted (v2 to v3) Annotation Transformation

We additionally performed a manual review of the LLM-based reformatting from v2 to v3 to assess how annotation structure is altered. Across the 20 cases, 6 files (30%) exhibit no change in the events, indicating that v2 annotations in these cases already satisfy the TTS criteria. For the remaining files, the number of events generally decreases, with reductions ranging from −1 to −106 events. Larger reductions are concentrated in MIMIC-IV cases (e.g., −73, −68, −106), whereas non-MIMIC cases typically show smaller changes (generally below −35 events).

The dominant transformations are consistent across files. First, fragmented event tokens are combined into coherent events, e.g., “taken”, “too many Xanax”→ “taken too many Xanax” and “Transferred”, “the floor” → “Transferred to floor”. Second, multiple tokens describing a single concept are consolidated, e.g., “substernal chest pain”, “exercise” → “substernal chest pain after exercise”. Third, incomplete or non-standalone fragments are removed, such as “kept”, “found”, or “presentation”, which do not correspond to valid clinical findings. Additional normalization includes abbreviation expansion (e.g., “HR” → “Heart rate”, “BP” → “Blood Pressure”) and construction of interpretable events from multi-token descriptions (e.g., “fell”, “riding skateboard”, “concrete” → “fell off board onto concrete landing”) .

Errors are infrequent and localized. Across the reviewed files, we identify 5 clear errors in total (0–2 per affected file, average ≈0.35 per file among modified cases), corresponding to approximately 0.23% of all events. These errors fall into two main categories: (i) incorrect span selection when multiple candidates exist (e.g., selecting “Axial MDCT images” is expanded to “Axial MDCT images of cervical spine” instead of “Axial MDCT images of the brain variant”; however this is not a case of hallucination since the exact same text appears in the note as well. This case happened only once), and (ii) inappropriate merging of events, either when timestamps differ or when concepts should remain separate (e.g., “nausea” and “vomiting” merged into a single event, or “discharged” combined with medication into “discharged medication OxycoDONE”) . Importantly, we do not observe hallucinated events; all outputs remain grounded in the original annotation content.

Overall, the reformatting primarily performs structured consolidation and cleanup, with errors occurring only in a small fraction of cases relative to the total number of transformations.

## Appendix E. Extended Results for Sensitivity Analyses

We provide full per-model sensitivity analysis results to complement the summary visualization presented in Figure 3 in the main text. While Figure 3 highlights the relative changes between subsets (i.e., Δ metrics), the tables in this section report the corresponding absolute values for each model and subset. Table E.1 presents the main results discussed in the paper, while Tables E.2 and E.3 report results under alternative versions of the manual reference annotations.

### Main results (v3 reformatted manual annotations).

Table E.1 provides the full per-model breakdown corresponding to Figure 3. These results are based on the final, fully aligned version of the manual annotations and serve as the primary reference for the sensitivity analysis discussed in the main text. As described there, the key trends include consistent improvements in AULTC for events supported by structured EHR evidence, strong gains in temporal ordering for central events (as reflected in both concordance and anchored concordance metrics), and a weak or inconsistent relationship between model-reported certainty and temporal ordering quality.

### Sensitivity analysis across gold-standard variants.

We replicate the sensitivity analysis presented in the main text under two alternative versions of the manual reference annotations (v1 and v2 defined in Appendix D.1), corresponding to the original released annotations and the rule-based cleaned version, respectively. Figures E.1 and E.2 summarize the subset-level effects, while the corresponding full tables (Tables E.2 and E.3) are provided alongside Table E.1. We focus on the same three factors as in the main analysis: structured EHR support (certain_EHR), model-reported certainty (certain), and central-event designation (is_central).

### Events supported by structured EHR evidence.

Across both v1 and v2, events with certain_EHR=1 consistently show improvements in AULTC across nearly all models, indicating better absolute timestamp accuracy when structured evidence is available. This pattern is already visible in v1 (Figure E.1) and becomes more consistent in v2 (Figure E.2), where the AULTC gains are more uniformly positive across models. Improvements in concordance and anchored concordance are more variable, with some models exhibiting modest gains and others showing neutral or slightly negative changes. Compared to the main results v3, the improvements in ordering metrics are weaker and less consistent, suggesting that better alignment between the gold standard and the TTS representation enhances the effectiveness of EHR-based temporal calibration for both absolute timing and relative ordering.

### Model-reported temporal certainty.

The certain flag exhibits inconsistent behavior across both v1 and v2. In both settings, AULTC tends to improve for certain=1 events, indicating that model confidence is somewhat aligned with absolute timestamp accuracy. However, concordance and anchored concordance frequently decrease for these events, particularly in v1, where negative Δ values for ordering metrics are common across models (Figure E.1). This pattern persists in v2, although with slightly reduced magnitude. Compared to v3, where this effect is clearer and more stable, the earlier versions show greater variability, suggesting that misalignment between annotation formats introduces additional noise that obscures the relationship between model confidence and temporal ordering.

**Table E.1:** Sensitivity analysis of multimodal timeline reconstruction across event subsets. Results are reported for events stratified by temporal certainty (certain), availability of structured EHR evidence (certain_EHR), and role in the reconstruction pipeline (is_central). We report event match rate (Match), temporal concordance (Conc.), anchored concordance (Anch.), anchored concordance with central anchors (Anch.-C), and AULTC.

Model	Subset	Match	Conc.	Anch.	Anch.-C	AULTC

DSR1	certain=0	0.5986	0.8186	0.8270	0.8449	0.7861
	certain=1	0.6261	0.7723	0.7771	0.8189	0.8288
	certain_EHR=0	0.6172	0.7751	0.7711	0.8023	0.8130
	certain_EHR=1	0.6441	0.8681	0.8182	0.9000	0.8699
	is_central=0	0.5961	0.7516	0.7688	0.8258	0.8227
	is_central=1	0.7596	0.8727	0.8125	0.8727	0.8105

DSv32	certain=0	0.6667	0.8202	0.7905	0.8333	0.7555
	certain=1	0.6399	0.7651	0.7869	0.8289	0.8367
	certain_EHR=0	0.6276	0.7845	0.8024	0.8246	0.8060
	certain_EHR=1	0.6944	0.7808	0.7700	0.8110	0.8582
	is_central=0	0.6276	0.7535	0.7808	0.8211	0.8296
	is_central=1	0.7279	0.8247	0.8211	0.8247	0.7839

GLM5	certain=0	0.5302	0.8788	0.8667	0.8844	0.7687
	certain=1	0.5910	0.8276	0.8258	0.8859	0.8515
	certain_EHR=0	0.5360	0.8137	0.8133	0.8566	0.7994
	certain_EHR=1	0.6553	0.8974	0.8668	0.9148	0.8822
	is_central=0	0.5534	0.7737	0.7816	0.8627	0.8370
	is_central=1	0.6627	0.9111	0.8761	0.9111	0.7971

GPT-OSS-120B	certain=0	0.5544	0.8218	0.8205	0.8440	0.7441
	certain=1	0.5689	0.7338	0.7594	0.8146	0.8145
	certain_EHR=0	0.5490	0.7710	0.7744	0.8341	0.7895
	certain_EHR=1	0.6340	0.8000	0.7832	0.8169	0.8413
	is_central=0	0.5319	0.7078	0.7447	0.8115	0.8081
	is_central=1	0.6541	0.8175	0.8311	0.8175	0.7865

KimiK2-Instruct	certain=0	0.4662	0.8333	0.7888	0.8598	0.7700
	certain=1	0.5875	0.7780	0.7655	0.7922	0.7710
	certain_EHR=0	0.5584	0.7772	0.7790	0.8043	0.7536
	certain_EHR=1	0.5919	0.7317	0.7549	0.7500	0.8101
	is_central=0	0.5653	0.7491	0.7662	0.7698	0.7693
	is_central=1	0.5798	0.9426	0.7819	0.9426	0.7769

Mistral-4-Small	certain=0	0.5009	0.5866	0.6134	0.6317	0.7106
	certain=1	0.4908	0.6076	0.6410	0.6250	0.7468
	certain_EHR=0	0.4935	0.5783	0.6021	0.5895	0.7229
	certain_EHR=1	0.5082	0.6375	0.7000	0.7264	0.7491
	is_central=0	0.4989	0.6080	0.6118	0.6142	0.7246
	is_central=1	0.4732	0.7417	0.6441	0.7417	0.7544

Qwen-3.5	certain=0	0.6135	1.0000	0.8709	0.9219	0.7649
	certain=1	0.6659	0.7508	0.7625	0.8154	0.8140
	certain_EHR=0	0.6355	0.7753	0.7676	0.7993	0.7917
	certain_EHR=1	0.7402	0.8071	0.7996	0.8377	0.8575
	is_central=0	0.6373	0.7238	0.7402	0.8068	0.8159
	is_central=1	0.7542	0.8602	0.8217	0.8602	0.7856

### Central events as temporal anchors.

The strongest and most consistent pattern across all annotation versions is observed for central events. In both v1 and v2, events with is_central=1 show clear improvements in concordance, anchored concordance, and anchored concordance (central) across most models (Figures E.1 and E.2). These gains are already visible in v1 and become slightly more pronounced and stable in v2. However, as in the main results, improvements in temporal ordering are not consistently accompanied by gains in AULTC, with several models showing neutral or negative changes in absolute timing. Compared to v3, the ordering improvements are present but less uniform, indicating that better representational alignment of the gold standard strengthens the role of central events as reliable temporal anchors.

### Comparison to main results.

Overall, the qualitative trends observed in the main text are already present in both v1 and v2, but become progressively clearer and more consistent as the gold standard is reformatted. In particular, (i) the AULTC gains from structured EHR evidence, (ii) the strong ordering improvements for central events, and (iii) the weak and inconsistent signal from model-reported certainty are all detectable in v1, sharpen in v2, and are most stable in v3. This progression indicates that the sensitivity analysis findings are robust to the choice of annotation reformatting that we have done, while also highlighting the importance of aligning the gold standard with the TTS representation for clearer and more reliable evaluation.

**Table E.2:** Sensitivity analysis results using the original released annotations v1. Metrics are defined as in Table E.1.

Model	Subset	Match	Conc.	Anch.	Anch.-C	AULTC

DSR1	certain=0	0.6721	0.8095	0.8130	0.8400	0.7812
	certain=1	0.7477	0.7606	0.7747	0.8441	0.8271
	certain_EHR=0	0.7291	0.7590	0.7576	0.8449	0.8130
	certain_EHR=1	0.7641	0.8167	0.8271	0.8750	0.8616
	is_central=0	0.7152	0.7269	0.7416	0.8410	0.8216
	is_central=1	0.8428	0.8667	0.8447	0.8667	0.8064

DSv32	certain=0	0.7110	0.8000	0.8067	0.8198	0.7573
	certain=1	0.7014	0.7760	0.7921	0.8348	0.8441
	certain_EHR=0	0.6780	0.7680	0.7885	0.8318	0.8149
	certain_EHR=1	0.7753	0.7992	0.7638	0.8402	0.8628
	is_central=0	0.6821	0.7483	0.7579	0.8183	0.8394
	is_central=1	0.8149	0.8759	0.8162	0.8759	0.7802

GLM5	certain=0	0.7118	0.8637	0.8234	0.9254	0.7640
	certain=1	0.7450	0.7860	0.8058	0.8407	0.8589
	certain_EHR=0	0.6757	0.7645	0.7892	0.8248	0.8008
	certain_EHR=1	0.8623	0.8310	0.8485	0.8727	0.8873
	is_central=0	0.7223	0.7554	0.7605	0.8364	0.8454
	is_central=1	0.8000	0.9424	0.8378	0.9424	0.7820

GPT-OSS-120B	certain=0	0.6863	0.8561	0.7878	0.8394	0.7560
	certain=1	0.6393	0.7902	0.8002	0.8418	0.8284
	certain_EHR=0	0.6354	0.7915	0.7894	0.8364	0.8051
	certain_EHR=1	0.7009	0.7810	0.7973	0.8227	0.8465
	is_central=0	0.5989	0.7548	0.7706	0.8311	0.8271
	is_central=1	0.7817	0.8384	0.8423	0.8384	0.7864

KimiK2-Instruct	certain=0	0.7102	0.8333	0.7895	0.8576	0.8077
	certain=1	0.6730	0.8037	0.7955	0.8034	0.7823
	certain_EHR=0	0.6540	0.7953	0.8085	0.8348	0.7716
	certain_EHR=1	0.7483	0.7259	0.7725	0.7601	0.8228
	is_central=0	0.6572	0.7705	0.7781	0.8025	0.7840
	is_central=1	0.7784	0.8944	0.8055	0.8944	0.7966

Mistral-4-Small	certain=0	0.6194	0.6006	0.6047	0.6688	0.7064
	certain=1	0.6673	0.6695	0.6718	0.6894	0.7683
	certain_EHR=0	0.6407	0.5858	0.6090	0.6409	0.7365
	certain_EHR=1	0.6474	0.5845	0.7047	0.7058	0.7368
	is_central=0	0.6400	0.6005	0.6080	0.6519	0.7360
	is_central=1	0.6579	0.7729	0.6768	0.7729	0.7415

Qwen-3.5	certain=0	0.5990	0.8860	0.8931	0.9214	0.7360
	certain=1	0.7370	0.7762	0.7848	0.8282	0.8153
	certain_EHR=0	0.6835	0.7824	0.7771	0.8412	0.7882
	certain_EHR=1	0.8647	0.8333	0.7972	0.8418	0.8664
	is_central=0	0.7066	0.7776	0.7717	0.8355	0.8167
	is_central=1	0.8028	0.8709	0.8366	0.8709	0.7770

**Table E.3:** Sensitivity analysis results using rule-based cleaned annotations v2. Metrics are defined as in Table E.1.

Model	Subset	Match	Conc.	Anch.	Anch.-C	AULTC

DSR1	certain=0	0.6294	0.7963	0.8259	0.8577	0.7906
	certain=1	0.6630	0.7792	0.7841	0.8312	0.8342
	certain_EHR=0	0.6559	0.8007	0.7852	0.8174	0.8197
	certain_EHR=1	0.6612	0.8452	0.7816	0.8413	0.8708
	is_central=0	0.6361	0.7695	0.7778	0.8343	0.8291
	is_central=1	0.7783	0.8627	0.8252	0.8627	0.8113

DSv32	certain=0	0.6596	0.7767	0.7919	0.8349	0.7567
	certain=1	0.6278	0.7497	0.7706	0.8189	0.8363
	certain_EHR=0	0.6106	0.7866	0.7813	0.8252	0.8071
	certain_EHR=1	0.7023	0.7400	0.7591	0.7824	0.8552
	is_central=0	0.6155	0.7331	0.7587	0.8089	0.8308
	is_central=1	0.7251	0.8366	0.8215	0.8366	0.7778

GLM5	certain=0	0.5734	0.8750	0.8679	0.8880	0.7628
	certain=1	0.6189	0.8210	0.8387	0.8953	0.8504
	certain_EHR=0	0.5749	0.8137	0.8224	0.8605	0.7977
	certain_EHR=1	0.6752	0.8935	0.8903	0.9129	0.8821
	is_central=0	0.5780	0.7717	0.7874	0.8688	0.8334
	is_central=1	0.7360	0.9153	0.8857	0.9153	0.8027

GPT-OSS-120B	certain=0	0.5644	0.8111	0.8205	0.8146	0.7429
	certain=1	0.5775	0.7494	0.7667	0.8225	0.8201
	certain_EHR=0	0.5573	0.7688	0.7725	0.8146	0.7959
	certain_EHR=1	0.6461	0.7652	0.7724	0.7761	0.8382
	is_central=0	0.5390	0.7201	0.7463	0.7901	0.8150
	is_central=1	0.6698	0.8111	0.8213	0.8111	0.7848

KimiK2-Instruct	certain=0	0.5097	0.8627	0.7932	0.8727	0.7683
	certain=1	0.6017	0.7715	0.7742	0.7884	0.7768
	certain_EHR=0	0.5763	0.8060	0.8013	0.8263	0.7592
	certain_EHR=1	0.6148	0.6734	0.7369	0.7500	0.8157
	is_central=0	0.5789	0.7544	0.7704	0.7729	0.7732
	is_central=1	0.6216	0.9083	0.7935	0.9083	0.7853

Mistral-4-Small	certain=0	0.5293	0.5782	0.6105	0.6320	0.7079
	certain=1	0.5399	0.6243	0.6465	0.6250	0.7508
	certain_EHR=0	0.5347	0.5856	0.6279	0.6313	0.7241
	certain_EHR=1	0.5323	0.6207	0.6818	0.7073	0.7478
	is_central=0	0.5352	0.6268	0.6256	0.6217	0.7266
	is_central=1	0.5268	0.7141	0.6401	0.7141	0.7428

Qwen-3.5	certain=0	0.6108	1.0000	0.8915	0.9219	0.7636
	certain=1	0.6689	0.7798	0.8045	0.8466	0.8179
	certain_EHR=0	0.6376	0.8169	0.8110	0.8471	0.7960
	certain_EHR=1	0.7468	0.8075	0.8024	0.8493	0.8602
	is_central=0	0.6408	0.7691	0.7841	0.8481	0.8206
	is_central=1	0.7614	0.8604	0.8521	0.8604	0.7841

## Appendix F. Extended results for ablation variants

**Table F.1:** Ablation results (event matching threshold = 0.1; (v3) version of manual annotations) for the three strongest models selected based on overall performance across event recovery and temporal metrics. Bold indicates the best value within each model block for a given metric. The “update central timeline only” condition yields a single final timeline and is therefore shown only in its multimodal form.

Model	Ablation	Event match rate	Concordance	AULTC

DeepSeek V3.2	Single-step			
	- Multimodal	0.420	0.781	0.819
	- Unimodal	0.420	0.758	0.809
	Update central timeline only			
	- Multimodal	0.548	0.749	0.817
	Update final timeline only			
	- Multimodal	**0.608**	0.731	0.809
	- Unimodal	0.606	0.739	0.805
	Update both central and final timeline			
	- Multimodal	0.588	**0.783**	**0.821**
	- Unimodal	0.588	0.772	0.814

DeepSeek R1	Single-step			
	- Multimodal	0.367	0.769	0.780
	- Unimodal	0.370	0.725	0.760
	Update central timeline only			
	- Multimodal	**0.586**	0.758	0.818
	Update final timeline only			
	- Multimodal	0.501	0.752	0.800
	- Unimodal	0.504	0.761	0.802
	Update both central and final timeline			
	- Multimodal	0.502	**0.788**	**0.820**
	- Unimodal	0.502	0.784	0.817

Qwen3.5-397B	Single-step			
	- Multimodal	0.368	0.722	0.783
	- Unimodal	0.368	0.705	0.768
	Update central timeline only			
	- Multimodal	**0.621**	0.784	0.813
	Update final timeline only			
	- Multimodal	0.564	**0 804**	**0 814**
	- Unimodal	0.564	0.799	0.803
	Update both central and final timeline			
	- Multimodal	0.580	0.759	0.809
	- Unimodal	0.580	0.776	0.802

Table F.1 and Figure F.1 extend the main-text ablation analysis to the three strongest models: DeepSeek V3.2, DeepSeek R1, and Qwen3.5–397B. A consistent pattern across all three models is that the single-step formulation is weakest. It occupies the lowest or near-lowest regions of the AULTC and concordance frontiers and underperforms the multistep variants in event recovery, indicating that factorizing timeline reconstruction around central events is beneficial beyond a single model.

The stage at which structured evidence is introduced, however, is more model-dependent. For DeepSeek V3.2, updating both the central and final timelines yields the best concordance and AULTC, while updating only the final timeline gives the highest event match rate. DeepSeek R1 shows a similar pattern: updating only the central timeline gives the strongest event match rate, whereas updating both stages yields the best temporal metrics. Qwen3.5–397B differs somewhat, with the central-only variant giving the highest event match rate but the final-only variant achieving the strongest concordance and AULTC. Thus, the appendix ablations suggest that the multistep scaffold is broadly useful, but the optimal point for multimodal calibration is not identical across models.

The threshold-sweep analyses reinforce that these conclusions are not specific to the threshold-0.1 operating point. Across a wide range of matching thresholds, the single-step variants remain clearly weaker than the multistep alternatives, while the stronger frontiers are achieved by different update schedules for different models. Taken together, these appendix results refine the main-text conclusion: reconstruction around central events is consistently valuable, and structured EHR evidence improves temporal quality most when introduced within a staged scaffolded pipeline, although the most effective stage for calibration varies by model.

## Appendix G. Extended results for multistep performance metrics

**Table G.1:** Comparison of model performance (event matching threshold = 0.1) under three versions of the manual gold standard: v1 (original released annotations), v2 (rule-based preprocessing), and v3 (rule-based + LLM reformatting). **Bold indicates the highest value in each metric/version column**. For event match rate, when unimodal and multimodal attain the same highest value, only the multimodal entry is bolded.

Modality	Model	Event match rate			Concordance			AULTC		

		v1	v2	v3	v1	v2	v3	v1	v2	v3

Multimodal (both central and final timelines updated)	DeepSeek R1	0.318	0.449	0.501	0.767	0.791	0.788	0.819	0.826	0.820
	DeepSeek V3.2	0.370	**0.508**	**0.588**	0.771	0.773	0.783	0.828	0.820	0.820
	GLM5	0.203	0.278	0.319	**0.807**	**0.816**	**0.812**	**0.836**	**0.827**	**0.829**
	KimiK2-Instruct	0.239	0.322	0.381	0.785	0.781	0.758	0.782	0.776	0.770
	Qwen3.5-397B	**0.395**	0.507	0.580	0.780	0.812	0.759	0.810	0.813	0.809
	GPT-OSS-120B	0.294	0.413	0.501	0.787	0.763	0.752	0.816	0.806	0.801
	Mistral-4-Small	0.167	0.240	0.278	0.608	0.635	0.629	0.740	0.728	0.728

Unimodal (text only)	DeepSeek R1	0.318	0.449	0.501	0.766	0.791	0.784	0.818	0.824	0.817
	DeepSeek V3.2	0.370	0.508	0.588	0.780	0.772	0.772	0.824	0.815	0.814
	GLM5	0.203	0.278	0.319	0.802	0.802	0.797	0.825	0.819	0.819
	KimiK2-Instruct	0.238	0.323	0.384	0.785	0.781	0.743	0.779	0.773	0.768
	Qwen3.5-397B	0.395	0.507	0.580	0.804	0.811	0.776	0.805	0.806	0.802
	GPT-OSS-120B	0.293	0.413	0.502	0.806	0.782	0.773	0.813	0.802	0.798
	Mistral-4-Small	0.167	0.240	0.278	0.614	0.617	0.622	0.737	0.723	0.721

### G.1. Performance metrics at event-matching threshold 0.1

Table G.1 shows how performance changes under the three versions of the manual gold standard: the original released annotations v1, a minimally cleaned rule-based version v2, and the final rule-based + LLM-reformatted version v3. The clearest pattern is that event match rate increases substantially and consistently from v1 to v2 to v3 across all models. For example, DeepSeek V3.2 improves from 0.370 to 0.508 to 0.588, Qwen3.5–397B improves from 0.395 to 0.507 to 0.580, and DeepSeek R1 improves from 0.318 to 0.449 to 0.501. This indicates that a substantial portion of the mismatch between model outputs and the original released annotations arises from representational differences between the concept-centric i2b2-style gold standard and the standalone event formulation.

The comparison between v1 and v2 shows that basic deterministic cleanup alone resolves a meaningful part of this mismatch. Removing section headers, exact duplicates, and other obvious non-events already yields a large improvement in event match rate. However, v3 consistently improves further over v2, indicating that semantic reformatting remains necessary even after trivial cleanup. This supports the view that the purpose of v3 is not merely cosmetic normalization, but a closer alignment of the manual reference with the TTS task definition.

In contrast, concordance and AULTC do not improve monotonically from v1 to v3. For many models, these temporal metrics are similar across versions, and in some cases they are slightly higher under v1 or v2 than under v3. This is expected because temporal metrics are computed only on matched events. As the reference becomes more TTS-compatible, more events become matchable, but those additional matches are often temporally harder. Thus, v3 broadens the evaluation set rather than simply making the benchmark easier.

Importantly, the main modality-level conclusions are robust across all three gold-standard versions. Event match rate remains nearly unchanged between unimodal and multimodal variants, whereas AULTC is consistently higher for the multimodal variant across all models and all three annotation versions. Concordance remains more model-dependent, but its overall pattern is similar to that observed in the main paper. Taken together, these results suggest that while absolute scores are sensitive to reference formatting, the central conclusion of the paper is stable: multimodal integration contributes primarily to temporal precision rather than to event recovery.

### G.2. Performance metrics for different thresholds

Figures G.1, G.2 and G.3 extend the comparison across manual gold-standard versions by plotting AULTC and concordance against event match rate as the event-matching threshold is varied from 0.01 to 0.50. Across all three manual versions, the clearest effect of moving from v1 to v2 to v3 is a rightward shift of the operating curves, indicating that increasingly TTS-compatible manual references allow substantially more predicted events to be matched. This pattern closely mirrors the appendix table, where event match rate increases consistently from v1 to v2 to v3 for all models. In contrast, the vertical position of the curves changes much less: AULTC and concordance remain in broadly similar ranges across versions, with mixed or smaller differences than those observed for event match rate. This suggests that gold-standard reformatting mainly broadens the set of events that can be aligned rather than uniformly improving temporal quality. Finally, the modality-level pattern remains stable across all versions: multimodal and unimodal variants usually occupy similar event-match ranges, while multimodal variants more often achieve slightly higher AULTC and, for some models, higher concordance. Taken together, the threshold sweeps corroborate the appendix table and support the same overall conclusion as the main paper: reformatting the manual reference has a strong effect on event recovery, whereas the benefit of multimodal integration is expressed primarily through improved temporal precision.
